# The effect of exercise on clinical pain: a systematic umbrella review and meta-meta-analysis

**DOI:** 10.1097/PR9.0000000000001455

**Published:** 2026-07-06

**Authors:** Ben Singh, Aaron Miatke, Dot Dumuid, Rachel Curtis, Kimberley Szeto, Kylie Dankiw, Emily Eglitis, Jasmine Petersen, Mason Zhou, Catherine Simpson, Lisa Matricciani, Felicity Braithwaite, Tasha Stanton, Carol Maher

**Affiliations:** a Alliance for Research in Exercise Nutrition and Activity (ARENA), Adelaide University, Adelaide, Australia; b IIMPACT, Adelaide University, Adelaide, Australia; c Persistent Pain Research Group, Hopwood Centre for Neurobiology, Lifelong Health Theme, South Australia Health and Medical Research Institute (SAHMRI), Adelaide, Australia

**Keywords:** Exercise, Pain, Physical activity, Chronic pain, Analgesia, Meta-analysis, Umbrella review

## Abstract

**Introduction:**

Exercise is recommended for managing pain, yet the consistency, magnitude, and certainty of effects across different pain conditions and exercises remain unclear.

**Objectives:**

This umbrella review aimed to synthesize the best available evidence on the analgesic effects of exercise by examining systematic reviews and meta-analyses of randomized controlled trials (RCTs).

**Methods:**

Eleven databases were systematically searched from inception to August 2024. Eligible studies included systematic reviews with meta-analyses of RCTs comparing exercise to control conditions, with pain as a primary or secondary outcome. Reviews without meta-analyses, those not involving RCTs, or those primarily focused on experimentally induced or laboratory pain were excluded. Two reviewers independently extracted data and assessed methodological quality using AMSTAR-2. Standardized mean differences in pain were synthesized using random-effects meta-meta-analysis. Certainty of evidence was evaluated using GRADE, with subgroup and sensitivity analyses.

**Results:**

A total of 157 systematic reviews comprising 2,736 RCTs and 221,279 participants were included. Exercise significantly reduced pain compared with controls (pooled standardized mean differences = −0.59; 95% CI, −0.65 to −0.53; *P* < 0.001). Effects were observed across both chronic and acute pain conditions, encompassing musculoskeletal, neurological, inflammatory, and cancer populations. Aerobic, resistance, yoga, Pilates, and tai chi were effective. Greater effects were observed in lower-intensity, shorter-duration (<12 weeks) programs. Sensitivity analyses supported the robustness of findings, and the overall GRADE certainty was moderate.

**Conclusion:**

This umbrella review provides compelling evidence that exercise offers consistent and clinically meaningful pain relief across diverse populations and conditions. Findings support the integration of even low-intensity, short-term exercise into routine pain management strategies.

## Abstract 2

Supplemental Digital Content is Available in the Text.

People with ongoing pain can benefit from starting low and going slow with exercise; it is safe, effective, and uniquely improves both pain and overall health.

## 1. Introduction

Chronic pain affects one in 5 adults globally^22,59^ and is a major contributor to disability and health care costs.^1,16,21,30,36,56,60,126^ Despite this enormous burden, effective, scalable, and safe treatment options remain limited. Pharmacological approaches such as opioids and NSAIDs are widely used but offer only modest relief and carry significant risks, particularly with long-term use.^26,37,43,150,155^ The opioid crisis has exposed the consequences of over-reliance on pharmaceutical solutions, with opioid-related deaths reaching epidemic proportions.^34^ As health care systems address these challenges, clinical guidelines increasingly recommend nondrug therapies including exercise.^173^

Yet in practice, exercise remains underused as a treatment for pain. It is rarely prescribed with the same precision as medication, and when it is recommended, the approach is often vague or poorly tailored.^55^ Patients commonly receive generic advice to “stay active” rather than evidence-based exercise prescriptions, whereas health care providers often lack confidence in recommending specific protocols.^14^ This gap persists despite compelling evidence that exercise can produce meaningful pain reductions across diverse conditions, working through anti-inflammatory effects, neuroimmune modulation, and central pain regulation.^113,157^ Unlike drugs, exercise offers sustained benefits, improved overall health, and minimal side effects when properly prescribed.

However, translation of this evidence into practice has been limited. The evidence, although extensive, is scattered across hundreds of condition-specific studies. A rheumatologist treating fibromyalgia might find strong evidence for yoga, whereas a physician managing osteoarthritis may find a review supporting resistance training. But neither has a clear picture of whether these represent fundamentally different therapeutic mechanisms or variations on the same theme. Previous umbrella reviews have not solved this problem—most focus on single conditions such as osteoarthritis,^98,166^ fibromyalgia,^6,18^ or dysmenorrhea,^53^ whereas others rely on narrative synthesis without quantitative analysis.^8^ This fragmentation leaves crucial questions unresolved for clinicians and guideline developers: to what extent exercise effects generalise across different pain conditions; which exercise modalities are most consistently associated with benefit; and how dose, duration, and intensity may be optimised to maximise analgesic effects.

Without clear answers, exercise prescription remains inconsistent, with well-intentioned but vague recommendations that may be suboptimal or even counterproductive. This represents a profound missed opportunity at a time when health care urgently needs safe, effective alternatives to pharmaceutical approaches.

Health care providers need definitive guidance, not just whether exercise helps, but which exercises work for which patients, and how to prescribe them effectively. The current evidence landscape of condition-specific reviews, with their overlapping studies and variable quality, cannot provide this clarity. What is required is a comprehensive synthesis with enough statistical power to identify consistent patterns and generate actionable clinical recommendations.

Comprehensive umbrella reviews with meta-analysis are particularly well suited to this challenge, as they provide a rigorous and scalable method for synthesising findings across multiple systematic reviews. By applying quantitative meta-analytic techniques, umbrella reviews allow for the aggregation of effect sizes across diverse populations, interventions, and conditions, offering greater statistical power and precision than narrative synthesis. This approach also enables formal assessment of methodological quality, publication bias, and the certainty of evidence, which are essential for translating research into practice. Crucially, meta-analysis can detect consistent patterns and subgroup effects, such as exercise mode, dose, and population characteristics, that would be difficult to identify within single-condition reviews. At a time when health care systems are seeking safe, scalable alternatives to pharmacological pain management, this level of comprehensive synthesis is critical for informing clinical guidelines and decision making.

This study addresses this need. We analyse data from 158 systematic reviews encompassing over 2,700 trials and nearly a quarter of a million participants. Rather than asking simply whether exercise reduces pain, we set out to examine how consistent these effects are across different populations and conditions, and to identify optimal prescribing parameters, using gold-standard approaches to assess the quality of the data and the confidence in the findings. By bringing together evidence from all pain conditions and exercise modalities, we aimed to provide the definitive foundation needed to make exercise a cornerstone of evidence-based pain management.

## 2. Methods

### 2.1. Protocol and registration

The protocol for this umbrella review was preregistered on PROSPERO (ID: CRD42024566338), and the results are presented in accordance with PRISMA guidelines.^132^

### 2.2. Selection criteria and search strategy

The inclusion criteria were developed using the population, intervention, comparison, outcomes, and study type (PICOS) framework as follows: Population: Any adult population aged 18 years and older (healthy and clinical). We included both healthy and clinical populations to capture pain outcomes across a broad spectrum, including acute or transient pain (eg, menstrual pain, exercise-induced pain) and to ensure a synthesis of evidence applicable to both prevention and management contexts. Reviews including children were excluded. Intervention: Reviews that evaluated exercise interventions were included. The following definition of exercise was used: “a type of physical activity consisting of planned, structured, and repetitive bodily movement done to improve or maintain a component of physical fitness.”^27^ We included structured physical activity meeting the definition of exercise (ie, planned, structured, and repetitive movement aimed at improving or maintaining physical fitness) and excluded broader terms such as sport, training, or treatment unless the intervention clearly aligned with this definition. Reviews were included if >75% of the included randomized controlled trials (RCTs) focused solely on exercise, including (but not limited to) aerobic exercise, resistance exercise, Pilates, yoga, tai chi, or qigong, which were not combined with any other intervention. Exercise interventions were included irrespective of exercise mode, supervision, delivery (eg, in-person or online), or dose (frequency, intensity and duration). We deliberately used the broader term “exercise” rather than “exercise therapy” to capture generalisable, nonclinically prescribed exercise interventions applicable to a wide range of populations and settings, including those not receiving treatment in a rehabilitation context, while still assessing pain as a clinical outcome. We considered pain reduction to refer specifically to reductions in pain intensity, as measured by standardized continuous self-report instruments (eg, Visual Analogue Scale [VAS], Numeric Rating Scale, and the pain subscale of the Western Ontario and McMaster Universities Osteoarthritis Index [WOMAC]), which were the most consistently reported pain outcomes across included reviews. Reviews were excluded if they included any non-RCTs, examined pain induced in a laboratory setting, or assessed only the acute effects of a single exercise session (ie, single-bout interventions), or therapeutic exercise protocols focused specifically on rehabilitation contexts (eg, poststroke, postjoint arthroplasty, shoulder rehabilitation, or postsurgical pain such as after total knee replacement). Comparator: Reviews were eligible if ≥75% of the included RCTs involved comparing either exercise to no intervention (including waitlist), usual care, nothing, a sham intervention or an equal attention nonexercise intervention arm, or a lower/lesser exercise intervention (eg, exercise intervention vs stretching). Outcomes: Pain was defined according to the International Association for the Study of Pain as “an unpleasant sensory and emotional experience associated with, or resembling that associated with, actual or potential tissue damage.”^86^ Eligible outcomes included any type of pain, such as general pain, low back pain, musculoskeletal pain, cancer-related pain, or headaches. Both acute and chronic pain presentations were eligible. Study type: Systematic reviews that included meta-analysis results of any pain outcome.

An electronic database search was performed using the following databases: CINAHL, The Cochrane Library, Embase via OVID, MEDLINE via OVID, Emcare via OVID, ProQuest Central, ProQuest Nursing and Allied Health Source, PsycINFO, Scopus, Sport Discus via Ebscohost, and Web of Science. Subject heading, keyword, and MeSH term searches for “systematic review,” “meta-analysis,” “exercise,” and “pain” were used (see Supplementary content 1 for the full search strategy, http://links.lww.com/PR9/A414). Database searches were limited to peer-reviewed journal articles published in English language from inception to 1 August 2024.

### 2.3. Data management and extraction

Search results were imported into EndNote X9 (Clarivate, Philadelphia, PA) for removal of duplicates. Results were then exported to Covidence (Veritas Health Innovation, Melbourne, Australia) for title/abstract and full-text screening, data extraction, and risk of bias assessment. All screening, data extraction, and risk of bias scoring were performed by 2 independent reviewers in duplicate. Discrepancies were resolved by discussion and consultation with a third reviewer if required.

A standardized Covidence (Veritas Health Innovation, Melbourne, Australia) extraction form was used to extract information on study details, sample characteristics, intervention details, outcomes, and results. Risk of bias was assessed using the AMSTAR-2 tool.^154^ The AMSTAR-2 consists of 16 items, each scored as either “Yes,” “Partial Yes,” or “No,” with 7 items considered “critical” and 9 items “non-critical.”^154^ The “critical” items are protocol registration; adequacy of search strategy; justification for excluding individual studies; risk of bias assessment; appropriateness of meta-analysis methods; use of risk of bias during interpretation; and assessment of publication bias. Reviews were rated as “high confidence” (no critical weakness and <3 noncritical weaknesses), “moderate” (1 critical weakness and <3 noncritical weaknesses), “low” (>1 critical weakness and <3 noncritical weaknesses), or “critically low” (>1 critical weakness and ≥3 noncritical weaknesses).^154^

### 2.4. Umbrella review synthesis methods

The amount of overlap of RCTs that were included across all eligible reviews was assessed using the Corrected Covered Area method.^74^ Corrected covered area ranges between 0% and 100%, with 0% indicating that every review consisted of entirely unique RCTs, whereas a score of 100% indicates that every review included the same RCTs. The following cut-offs were used for CCA: ≤5%: “slight overlap”; 6% to 10%: “moderate”; 11% to 15%: “high”; and >15%: “very high” overlap.^135^

Meta-analyses for pain were performed by pooling the effect sizes and 95% confidence intervals (CIs) reported in each review using the immediate postintervention time point, using a random effects model. Meta-analyses were conducted using standardized effect sizes (ie, standardized mean difference, SMD) as the primary effect measure. When effect sizes or 95% CIs were not reported, authors were contacted to request the necessary data. If no response was received, reviews were excluded from the quantitative synthesis. Results of meta-analyses were displayed visually using forest plots. Subgroup analyses were performed for type of pain (acute and chronic) and population, exercise mode (aerobic, aquatic, dance, HIIT, mind body [various], mixed-mode, Pilates, resistance, tai chi, telehealth, exercise, virtual reality, and yoga), intervention duration (<12 weeks, ≥12 weeks), intensity (low, moderate-to-vigorous), sessions per week (1–2, 3 or more), session duration (≤60 minutes, >60 minutes), weekly duration (<120 minutes, ≥120 minutes), and AMSTAR-2 score (critically low, low, moderate, high). Exercise intensity classifications were extracted as reported in each included review. Where specified, low-intensity exercise typically referred to light activities requiring minimal cardiovascular effort (eg, tai chi, yoga). Given the variability in how intensity was defined or measured across reviews, we retained the original authors' terminology and categorized intensity accordingly. The proportion of the overall outcome attributed to variability was measured using the I^2^ statistic.^40^ The following values were used to determine the level of heterogeneity: 0% to 29% = no heterogeneity; 30% to 49% = moderate heterogeneity; 50% to 74% = substantial heterogeneity; and 75% to 100% = considerable heterogeneity.^79^ The following classifications were used to quantify the size of effects: small effect: <0.20; medium effect: 0.20 to 0.50; and large effect: >0.50.^100^ A *P*-value of <0.05 was considered statistically significant. Publication bias was assessed using funnel plots of the pooled effects from the included reviews, and assessing for the presence of asymmetries or missing sections.^46^ All meta-analyses were performed using Stata/MP (v16, Stata Corp, College Station, TX).

The GRADE (Grading of Recommendations, Assessment, Development and Evaluations)^61^ approach was used to rate the certainty of evidence for each outcome, considering 5 key domains: risk of bias, inconsistency, indirectness, imprecision, and publication bias. Based on these criteria, outcomes related to pain were rated as having high, moderate, low, or very low certainty.

### 2.5. Deviations from protocol

An exploratory subgroup analysis comparing reviews focused on acute versus chronic pain was added in response to peer-review feedback.

## 3. Results

**Figure 1.**
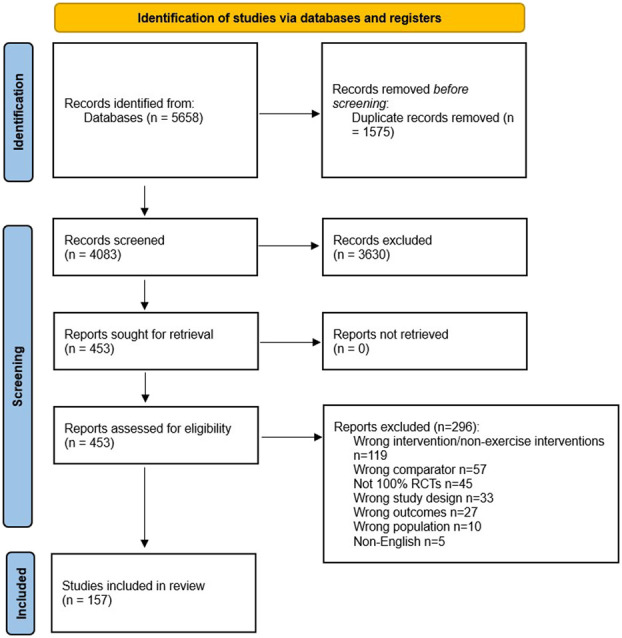
PRISMA flowchart.

A total of 5,658 search results were identified after a search of databases, of which 158 reviews were eligible and included. The PRISMA flowchart including reasons for exclusions is shown in Figure 1. A full list of reasons for full-text exclusions (n = 296) is shown in supplemental digital content 2 (http://links.lww.com/PR9/A414). The 157 included reviews comprised 2736 RCTs and a total of 221,279 participants. Publication years of the included reviews ranged from 2002^23^ to 2024.^9,12,19,39,42,47,54,68,116,121,140,159,170,176,195^ The overall CCA was 0.6%, indicating slight overlap of the same RCTs.

An overview of age groups, populations, and exercise intervention characteristics are shown in supplemental digital content 3 (http://links.lww.com/PR9/A414). Mean participant age ranged between 20 and 79 years, and most reviews (n = 135) included both female and male participants (females only, n = 22^38,39,42,49,50,68,82,94,106,108,118,139,145,147,151,156,175,180,184,190,193^). The populations and types of pain evaluated involved knee osteoarthritis (n = 28^2,7,31,51,62,64,83,90,91,103,107,110,116,117,123,133,140,146,159–161,164,174,176,187,191,196,200^), low back pain (n = 22^3,5,13,58,72,73,76,81,95,112,125,134,142,143,167,179,182,192,195,200,202,203^), osteoarthritis (mixed, n = 18^19,29,45,47,48,67,80,84,88,89,92,93,102,121,153,169,183,195^), fibromyalgia (n = 16^4,23–25,33,35,38,44,71,101,128,144,147,168,170,180^), cancer (n = 14^9,32,63,68,82,85,105,118,129,136,139,145,162,186^), chronic musculoskeletal pain (n = 11^12,54,65,66,97,124,131,137,152,172,178^), osteoporosis and osteopenia (n = 7^106,108,109,138,177,184,198^), pregnancy-related (n = 7^42,49,50,151,156,190,193^), rheumatoid arthritis (n = 6^10,11,20,158,188,189^), hip arthritis (n = 5^52,69,77,127,163^), migraine and headache (n = 4^99,115,165,181^), neck pain (n = 3^15,96,111^), neurologic related pain (n = 3^17,41,67^), type 2 diabetes (n = 2^141,201^), and various others (n = 11,^39,70,94,104,108,119,120,130,171,175,185^ see supplemental digital content 3, http://links.lww.com/PR9/A414).

The types of exercise evaluated across the reviews involved mixed mode (n = 69^2,4,7,9,12,13,15,20,23–25,31,35,41,42,47,48,50–52,63,64,68,71,72,76,77,80,84,85,90,91,93,108,111,119–121,123,124,127,129–131,133,136,137,143–146,151,153,157,159,160–165,167,171,179,184,186,187,194,195^), tai chi (n = 17^32,33,65,66,83,92,97,103,118,141,142,175,183,185,192,198,201^), yoga (n = 15^17,19,81,82,94,95,102,104,115,116,175,181,190,194,202^), mind–body (various, mixed types of mind–body such as yoga and tai chi, included in the same review, n = 15^44,54,58,96,101,106,107,140,159,178,196,197,199,200,203^), resistance (n = 13^10,29,38,69,70,109,110,114,138,139,148,168,170^), aerobic (n = 8^3,5,11,99,105,117,152,188^), Pilates (n = 7^39,49,112,125,134,182,190^), aquatic (n = 6^45,67,73,88,169,172^), qigong (n = 4^64,89,177,191^), exercise and virtual reality (n = 2^176,180^), and dance (n = 1^128^). Interventions ranged between 1 week and 240 weeks. Results of the AMSTAR-2 scores are shown in supplemental digital content 4 (http://links.lww.com/PR9/A414). Most reviews had a low AMSTAR-2 rating (n = 93,^2,9,12,13,17,19,23,29,31–33,38,41,44,45,49–51,58,62,64,69,70,73,76,80,83–85,89,91,94–97,99,105,107,111,114–116,119,121,123,124,128,129,131,133,136,138–143,147,151,153,156,158,159,163,165,170,173,175–181,184–187,190–203^), and the remaining had a critically low (n = 54,^3–5,10,11,15,25,35,39,42,47,48,63,65–67,71,72,77,81,82,88,90,92,93101–103,106,108–110,112,117,118,120,125,127,134,137,144,146,152,160–162,164,167,168,170,171,183,188,203^) or high (n = 10^7,20,24,52,54,68,104,130,145,182^) rating.

### 3.1. Meta-analyses results

#### 3.1.1. Pain (overall)

**Figure 2.**
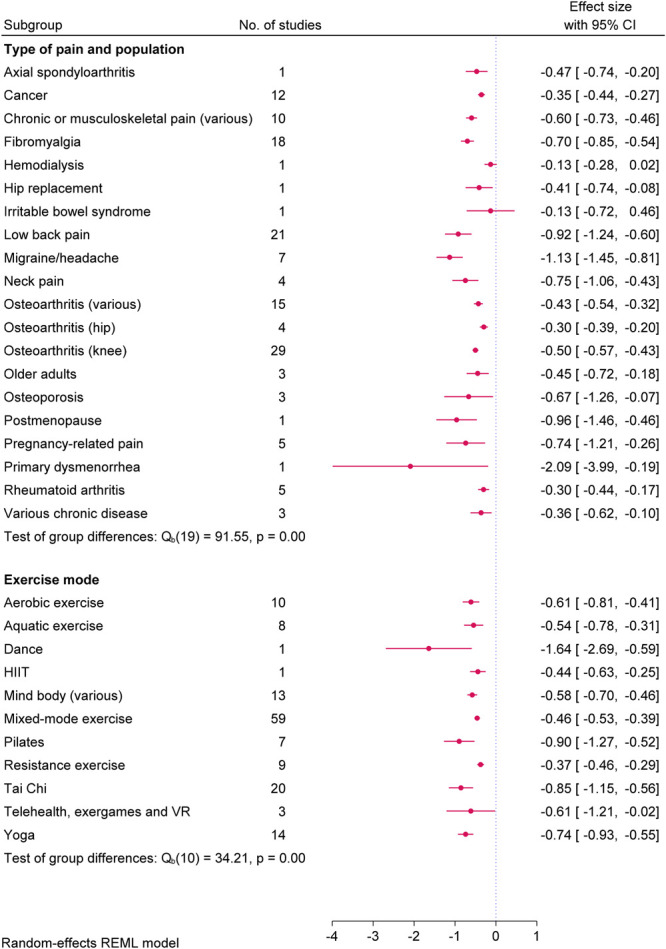
Meta-analysis of populations and types of pain, and exercise modes.

Pooled analysis of 144 meta-analyses (n = 197,392 participants) using SMD showed a large and significant reduction in overall pain with exercise immediately postintervention (SMD = −0.59, 95% CI = −0.65 to −0.53, I^2^ = 88.17%, *P* < 0.01, supplemental digital content 5, http://links.lww.com/PR9/A414). Results of meta-analysis by population and type of pain and exercise mode are shown in Figure 2.

#### 3.1.2. Population and type of pain

Significant reductions in pain were observed for axial spondylarthritis, cancer, chronic or musculoskeletal pain (nonspecific), fibromyalgia, hip replacement, low back pain, migraine and headache, neck pain, osteoarthritis (various, knee, hip), idiopathic pain in older adults, osteoporosis, postmenopausal, pregnancy-related pain, primary dysmenorrhea, rheumatoid arthritis, and various chronic diseases (including neurologic diseases) (SMD range = −2.09 to −0.30, all *P* < 0.05). The largest reductions in pain were observed for primary dysmenorrhea (SMD = −2.09 [95% CI = −3.99 to −0.19]), migraine/headache (SMD = −1.13 [95% CI = −1.45 to −0.81]), and postmenopausal (SMD = −0.96, [95% CI = −1.46 to −0.46]).

#### 3.1.3. Exercise mode

Significant, large reductions in pain were observed for various modes of exercise, including aerobic, aquatic, dance, HIIT, mind body (various), mixed-mode, Pilates, resistance, tai chi, telehealth, exergames and VR, and yoga (SMD range = −1.64 to −0.37, all *P* < 0.05). The largest reduction in pain was observed for dance (SMD = −1.64 [95% CI = −2.69 to −0.59]), Pilates (SMD = −0.90 [95% CI = −1.27 to −0.52]), and tai chi (SMD = −0.85 [95% CI = −1.15 to −0.56]).

There were sufficient MD data for the following instruments: VAS 1 to 10 scale and 1 to 100 scale, WOMAC, and Short Form-36 Health Survey (SF-36, Table 1). MD effect sizes for each instrument were VAS (1–10): −1.10 (95% CI = −1.48 to −0.71), VAS (1–100 scale): −10.89 (95% CI = −13.93 to −7.85), WOMAC Score: −6.70 (95% CI = −17.96 to 4.56), and SF-36—Pain: 2.97 (95% CI = −0.83 to 6.76, positive scores represent less pain).

**Table 1:** Mean difference (MD) effects for pain.

	No. of studies	MD	95% CI	I^2^ (%)	*P*
Visual analogue scale (1–10)	25	−1.10	−1.48 to −0.71	92.65	<0.01
Visual analogue scale (1–100)	8	−10.89	−13.93 to −7.85	71.35	<0.01
WOMAC score	2	−6.70	−17.96 to 4.56	93.03	0.24
SF-36—pain*	6	2.97	−0.83 to 6.76	89.04	0.13

* Positive values represent reduction in pain.

MD, mean difference; SF-36, Short Form [36] Health Survey; WOMAC, Western Ontario and McMaster Universities Arthritis Index.

### 3.2. Subgroup analyses

Results of subgroup analyses are shown in supplemental digital content 7 (http://links.lww.com/PR9/A414).

#### 3.2.1. Type of pain

The effects of exercise did not differ between acute (SMD = −0.87 [95% CI: −1.21 to −0.53]) compared with chronic pain (SMD = −0.57 [95% CI: −0.64 to −0.51], test of subgroup differences: Qb(1) = 2.76, *P* = 0.10).

#### 3.2.2. Intervention duration

Greater effects were observed for interventions that were less than 12 weeks (SMD = −0.59 [95% CI: −0.78 to −0.41]) compared with 12 weeks or more (SMD = −0.37, [95% CI: −0.45 to −0.29], test of subgroup differences: Qb(1) = 4.61, *P* = 0.03).

#### 3.2.3. Exercise intensity

Greater effects were observed for low intensity exercise (SMD = −0.77 [95% CI = −0.90 to −0.64]) compared with moderate-to-vigorous intensity (SMD = −0.47 [95% CI = −0.52 to −0.41], test of subgroup differences: Qb(1) = 17.80, *P* < 0.01).

#### 3.2.4. Sessions per week and session duration

No subgroup differences were observed between 1 and 2 compared with 3+ sessions per week (test of subgroup differences: Qb(1) = 0.13, *P* = 0.72), and when performing <60-minute sessions compared with >60-minute sessions (test of subgroup differences: Qb(1) = 1.25, *P* = 0.26).

#### 3.2.5. Weekly duration

Greater reductions in pain were observed when exercising <120 minutes per week (SMD = −0.90 [95% CI = −1.80 to 0.00]) compared with ≥120 minutes per week (SMD = 0.58 [95% CI = −0.27 to 1.42], test of subgroup differences: Q_b_(1) = 5.48, *P* = 0.02).

#### 3.2.6. AMSTAR-2 score

A significant difference was observed between studies rated as high (SMD = −0.40 [95% CI = −0.48 to −0.33]), low (SMD = −0.64 [95% CI = −0.71 to −0.56]), and critically low (SMD = −0.57 [95% CI = −0.70 to −0.43], test of subgroup differences: Qb(2) = 18.73, *P* < 0.01).

#### 3.2.7. Sensitivity analyses and publication bias

Visual inspection of the funnel plot for pain (supplemental digital content 6, http://links.lww.com/PR9/A414) displayed a degree of asymmetry, with a gap in the bottom right quadrant suggesting a lack of smaller studies reporting negative effect sizes, a pattern indicative of potential publication bias. To account for this potential bias, we calculated the estimated true effect size (theta, θ), which corrects for possible missing studies. The corrected effect size (θ = −0.43).

#### 3.2.8. Grading of Recommendations, Assessment, Development, and Evaluations assessment

Based on the GRADE framework, the certainty of evidence for the effect of exercise on overall pain was rated as moderate (supplemental digital content 8, http://links.lww.com/PR9/A414). This rating reflects the inclusion of a large number of RCTs summarised across systematic reviews (level 1 evidence), consistent findings across diverse populations and exercise types, and a large effect size (SMD = −0.59). However, the certainty was downgraded one level because of concerns about methodological quality (AMSTAR-2 indicated many included reviews were low or critically low quality) and potential publication bias (asymmetry in funnel plots). There was no serious concern regarding indirectness, imprecision, or inconsistency.

## 4. Discussion

This is the most comprehensive synthesis of exercise for pain relief to date, and the findings are clear: exercise reduces pain across a broad spectrum of conditions, with benefits that are both statistically significant and clinically meaningful, comparable to, or exceeding, many pharmacological therapies. We observed a large and statistically significant overall effect (SMD = −0.59), with consistent pain reduction across all exercise types and populations, ranging from primary dysmenorrhea (SMD = −2.09) to migraine/headache (SMD = −1.13). Notably, exercise was associated with large reductions in acute pain (SMD = −0.87), further supporting its analgesic potential beyond chronic pain populations. Our subgroup analyses suggested that programs that were shorter (<12 weeks), lower in intensity, and under 120 minutes per week yielded the largest benefits. Together, these findings indicate that even modest amounts of exercise can effectively reduce pain, providing clinicians with considerable flexibility and increased confidence to prescribe exercise as a core, scalable component of pain management across diverse conditions.

Previous umbrella reviews have focused narrowly on individual conditions such as osteoarthritis, fibromyalgia, or dysmenorrhea, or relied on narrative synthesis without pooled analyses.^6,8,18,53,98,166^ In contrast, our review integrates findings from 157 systematic reviews encompassing 2736 randomised controlled trials and over 220,000 participants. By drawing on such a diverse and extensive evidence base, this review enables the identification of generalisable patterns and principles that can inform more practical, flexible, and condition-agnostic exercise prescriptions. These findings directly address the gap between research and clinical application, offering a unifying framework for incorporating exercise into pain management across a wide range of health care settings.

Although the pooled effect size observed (SMD = −0.59) is considered large and is comparable to, or greater than, the effects observed for many pharmacological pain treatments, it is important to note that medications typically yield modest improvements in persistent pain.^26^ Exercise should not be considered a universal solution or expected to eliminate pain entirely. Its efficacy will depend on individual factors such as the underlying pain condition, level of physical function, and adherence.^57^ However, although medication has substantial risks when used long term,^87^ exercise provides a sustainable, low-risk option with additional psychological and functional benefits. Accordingly, exercise should be positioned as a foundational component of multimodal, patient-centred pain care.

This review builds on and significantly extends the current literature, offering the first quantitative synthesis of exercise's analgesic effects across the full spectrum of pain conditions. Prior umbrella reviews have typically been restricted to specific conditions, such as osteoarthritis^166^ or migraine,^78^ or relied on narrative synthesis without pooled effect sizes. Our findings for osteoarthritis (SMD = −0.50 to −0.30 across 48 meta-analyses) and migraine/headache (SMD = −1.13 across 7 meta-analyses) are consistent with prior condition-specific reviews, reinforcing their conclusions. However, by aggregating data across 158 reviews and 222,585 participants, we demonstrate that the benefits of exercise are not limited to isolated conditions but generalisable across pain types. Our present findings showed that across pain types, exercise demonstrated substantial analgesic effects, with reductions for acute (SMD = −0.87) and chronic pain (SMD = −0.57), indicating broadly consistent benefits of exercise across pain durations. This broader perspective is crucial for informing clinical guidelines and justifying the integration of exercise into routine care. Our GRADE assessment rated the overall certainty of evidence as moderate, supporting confidence in the findings, while also acknowledging limitations related to review quality and potential publication bias. The relatively low overlap of primary studies between reviews reflects the broad inclusion criteria of this umbrella review, which covered varied populations, pain types, and exercise modalities. As a result, most systematic reviews addressed distinct subgroups, minimising duplication of primary studies.

The strong effectiveness across diverse exercise modalities offers a key advantage for clinical practice: flexibility. Whether through aerobic training, resistance exercises, or mind–body approaches such as tai chi and Pilates, exercise consistently reduced pain, allowing clinicians to tailor prescriptions to patient preferences, capabilities, and contexts. The particularly large effects observed for mind–body modalities align with emerging evidence that these approaches may target both physical and psychological dimensions of pain. This is especially relevant for patients with central sensitisation, who may benefit from gentle, integrative forms of movement that also support relaxation and body awareness. Further, the large effect observed for dance (SMD = −1.64) should be interpreted with caution, as it was derived from a single systematic review focused on fibromyalgia. Although dance-based interventions may offer promise for this population, further research across broader groups is needed before clinical recommendations can be made. This applies similarly to other exercise modalities, where effect sizes may reflect evidence from a limited number of systematic reviews and should, therefore, be considered within the context of the available evidence base.

These findings suggest a need to reconsider traditional exercise prescriptions for pain. Low-intensity exercise produced greater pain relief than moderate-to-vigorous activity, and shorter programs (<12 weeks) showed larger effects than longer ones. However, this should not be interpreted as evidence that exercise becomes less effective over time. A more likely explanation is that people tend to do less exercise beyond the structured intervention period. Many trials provide a fixed-duration program and then withdraw formal support, assuming participants will continue independently, something that is often not sustained in practice. As such, the smaller effects observed in longer-duration programs likely reflect reduced exercise exposure, not diminishing benefits. These findings support starting with manageable, lower-dose programs to build confidence, promote adherence, and reduce fear of symptom flare-ups, key barriers for people with chronic pain. Although early adaptations to low-intensity exercise may drive short-term benefits, ongoing support and progression may be needed to sustain improvements over time. It is important to note that the largest pain reductions observed for primary dysmenorrhea (SMD = −2.09), migraine/headache (SMD = −1.13), and postmenopausal-related pain (SMD = −0.96) were each derived from single systematic reviews. Although these reviews included multiple trials and used standard meta-analytic methods, findings from a single review should be interpreted with some caution. These conditions appear to respond particularly well to exercise, but further high-quality reviews and primary studies are needed to confirm and extend these results.

The finding that programs under 120 minutes per week were more effective than higher weekly-duration programs has important practical implications. Many current exercise guidelines recommend 150 minutes per week or more, which can feel unattainable for people living with chronic pain. Our results suggest that lower-duration programs may not only be more realistic but also more effective, particularly for those who are time-poor or fearful of exacerbating symptoms. Similarly, the absence of significant differences between 1 and 2 vs 3+ sessions per week offers valuable flexibility, allowing clinicians to design exercise programs that better align with individual preferences, routines, and capacity for change.

Our findings highlight the significant analgesic effects of exercise and raise important questions about how these benefits are achieved. Although exercise influences multiple biological systems, 4 key mechanisms appear most relevant. First, exercise stimulates the release of endogenous opioids (eg, endorphins), which act as natural painkillers and increase pain tolerance.^149^ Second, it enhances the production of neurotransmitters such as serotonin and norepinephrine, both of which play crucial roles in mood and pain modulation.^149^ Third, exercise reduces systemic inflammation, a common driver of chronic pain, by modulating immune function.^157^ Fourth, exercise activates the endocannabinoid system, another intrinsic pain-regulating network, by increasing levels of endocannabinoids such as anandamide, which bind to cannabinoid receptors and help modulate pain perception, mood, and inflammation.^122^ This mechanism is believed to contribute to exercise-induced hypoalgesia, particularly in chronic pain populations.^122^ Together, these central and peripheral effects help explain the consistent pain-reducing benefits observed across diverse conditions and populations. Understanding these core mechanisms reinforces the rationale for including exercise as a foundational component of modern pain management strategies. Beyond its analgesic effects, exercise offers a range of well-documented health benefits, including improvements in cardiovascular health, strength, flexibility, balance, and endurance, that can contribute to reduced pain sensitivity and improved quality of life. These systemic and biomechanical adaptations may help individuals better tolerate physical activity and reduce activity-related pain over time.^28,75^ These additional benefits reinforce exercise as a safe, cost-effective, and holistic approach to managing chronic pain.

### 4.1. Strengths and limitations

This umbrella review has several key strengths that distinguish it from previous syntheses in this field. First, by including only systematic reviews of randomized controlled trials, we focused on the highest level of experimental evidence. Second, our inclusion of all pain conditions and exercise modalities provides the most comprehensive and generalisable picture to date of how exercise can reduce pain. With data from 222,585 participants, this synthesis draws on an exceptionally large evidence base. Importantly, we found minimal overlap in primary studies across reviews (CCA of 0.6%), increasing confidence that the overall findings are not unduly influenced by duplication or overly represented trials.

Our detailed subgroup analyses also offer practical insights for tailoring exercise prescriptions to different patient groups and contexts. Although many included reviews were rated as low in quality, our sensitivity analyses showed that meaningful analgesic effects were still evident in reviews rated as high quality (SMD = −0.40). Similarly, adjusted analyses accounting for publication bias produced only a modest attenuation of effect size (SMD = −0.43), reinforcing the robustness of the overall findings. Although the general conclusion that exercise can reduce pain has been reported previously, the present umbrella review provides high-level quantitative synthesis across a large and fragmented literature, in which individual reviews have reported mixed and often null findings. By integrating this evidence, our findings help clarify the overall magnitude and consistency of analgesic effects associated with exercise.

Further limitations are that the included studies used different ways of measuring pain and describing exercise programs, making it challenging to compare results directly. Beyond broad acute/chronic categories, we were unable to distinguish more granular pain subtypes. Many studies also did not follow up with participants long term, so we know less about how long the benefits of exercise last. In addition, studies varied in how they reported whether participants stuck to their exercise programs, making it difficult to determine the optimal level of exercise adherence. As with all umbrella reviews, we appraised methodological quality at the level of the included systematic reviews rather than the original trials. This is a recognised limitation of the design and should be considered when interpreting findings. It is also important to consider that the number and quality of reviews and underlying trials varied across conditions, and effect sizes should be interpreted alongside CIs and GRADE ratings, rather than based on study counts alone. Differences in effect sizes across pain measurement tools (eg, VAS, WOMAC, SF-36) likely reflect variations in scale sensitivity, construct focus, and scoring range. For instance, VAS provides a direct measure of pain intensity and may be more responsive to short-term change, whereas composite instruments such as the SF-36 Pain subscale assess broader impacts of pain on functioning, which may yield smaller or more variable effect sizes. These differences highlight the importance of considering the measurement tool when interpreting intervention outcomes.

The breadth of this synthesis provides, first, strong evidence of effectiveness, and second, actionable insights for clinical application and research priorities. One of the most important findings is that shorter, low-intensity exercise programs (<12 weeks, <120 min/wk) consistently produced the largest pain reductions. These results challenge assumptions that longer or more intense programs are necessarily better. A likely explanation is that shorter programs may achieve better adherence, particularly among individuals living with pain, who may be discouraged by longer commitments or fears of exacerbating symptoms. This highlights the value of starting with accessible, manageable interventions. It also suggests a need for future studies examining how to support sustained engagement and gradual progression over time.

The very large effect sizes observed for certain conditions, such as primary dysmenorrhea (SMD = −2.09) and migraine (SMD = −1.13), also warrant further investigation. These condition-specific patterns may reflect underlying biological or behavioural mechanisms that could guide more targeted and effective prescriptions.

Beyond what works, future research should focus on how exercise programs can be best delivered and implemented in practice. Key questions include the role of supervision, setting (eg, home-based vs clinic-based), and delivery mode (eg, digital, group, individual). Our findings suggest that programs do not need to be lengthy or intense to be effective, creating opportunities to design scalable, low-burden interventions that fit within routine clinical care.

Finally, very few studies included long-term follow-up, limiting our ability to assess the durability of exercise benefits. There is a need for trials that explore maintenance strategies beyond the intervention period and particularly approaches that reflect real-world implementation constraints. Taken together, these priorities signal a shift in the field; it is clear that we no longer need research asking whether exercise works, instead, we need research addressing how best to deliver it and sustain it in diverse, real-world populations.

## 5. Conclusion

This umbrella review provides robust evidence supporting the effectiveness of exercise for managing a wide range of pain conditions. Our findings suggest that relatively brief, low-intensity programs, often perceived as more achievable by people living with chronic pain, are associated with greater pain reductions on average. However, these patterns reflect trends across diverse studies and should not be interpreted as prescriptive. Rather, they underscore the importance of starting with accessible, lower-dose programs that can be adjusted based on individual needs, preferences, and progression. Given the consistent benefits observed across exercise types and populations, clinicians are encouraged to integrate exercise as a core component of multimodal pain care. These findings reinforce the role of exercise as a safe, adaptable, and patient-centred option, particularly valuable in addressing the limitations of pharmacological pain management. Future research should focus on how to best individualise, deliver, and sustain effective exercise interventions in real-world clinical settings.

## Disclosures

The authors have no conflict of interest to declare.

## Supplemental digital content

Supplemental digital content associated with this article can be found online at http://links.lww.com/PR9/A414.
