# Supplementary material for: The effect of exercise on clinical pain: a systematic umbrella review and meta-meta-analysis
Source: Pain Rep. 2026 Jul 6;11(4):e1455. doi: 10.1097/PR9.0000000000001455 (PMC13340705; doi:10.1097/PR9.0000000000001455)
Supplement: Supplementary file 1 [file painreports-11-e1455-s001.pdf]

Contents

Supplementary content 1. Search strategy. 2
Supplementary content 2. List of reasons on exclusion for all full texts (n=296) 3
Supplementary content 3. Overview of all included systematic reviews and meta-analyses (n=158) 15
Supplementary content 4. AMSTAR-2 risk of bias and study quality ratings. 26
Supplementary content 5. Overall meta-analysis results for pain. 33
Supplementary content 6. Funnel plots for pain. 34
Supplementary content 7. Subgroup effects for physical activity parameters for pain 35
Supplementary content 8. Overview of GRADE ratings for pain. 36

Supplementary content 1. Search strategy.

Field	Search terms
Exercise	"Exercise"[Mesh] OR "Exercise Therapy"[Mesh] OR "Physical Education and Training"[Mesh] OR "Sports"[Mesh] OR exercise*[tiab] OR "physical activity"[tiab] OR "physical activities"[tiab] OR sport*[tiab] OR "physical education"[tiab] OR "physical training"[tiab] OR "resistance training"[tiab] OR "strength training"[tiab] OR "aerobic training"[tiab] OR "anaerobic training"[tiab] OR "endurance training"[tiab] OR "high-intensity interval training"[tiab] OR "plyometric training"[tiab] OR "circuit training"[tiab] OR yoga[tiab] OR pilates[tiab] OR "tai chi"[tiab] OR "qigong"[tiab] OR swimming[tiab] OR running[tiab] OR jogging[tiab] OR walking[tiab] OR cycling[tiab] OR "weight lifting"[tiab] OR "weight training"[tiab]
Pain	"Pain"[Mesh] OR "Chronic Pain"[Mesh] OR "Pain Management"[Mesh] OR pain*[tiab] OR analgesia[tiab] OR analgesic*[tiab] OR nociception[tiab] OR hyperalgesia[tiab] OR allodynia[tiab] OR "pain relief"[tiab] OR "pain reduction"[tiab] OR "pain management"[tiab]
Systematic Reviews and Meta-analyses	# Concept 3: Systematic Reviews and Meta-analyses "Systematic Review"[Publication Type] OR "Meta-Analysis"[Publication Type] OR "Systematic Reviews as Topic"[Mesh] OR "Meta-Analysis as Topic"[Mesh] OR "systematic review"[tiab] OR "systematic reviews"[tiab] OR "systematic literature review"[tiab] OR "systematic literature reviews"[tiab] OR "meta-analysis"[tiab] OR "meta-analyses"[tiab] OR "meta analysis"[tiab] OR "meta analyses"[tiab] OR metaanalysis[tiab] OR metaanalyses[tiab] OR "pooled analysis"[tiab] OR "pooled analyses"[tiab] OR "umbrella review"[tiab] OR "umbrella reviews"[tiab]
	# Combined Search Strategy<br>(#1 AND #2 AND #3)
	# Filters<br>Filters: Humans[Mesh]
	# Date Range<br>Inception to August 1st, 2024.

Supplementary content 2. List of reasons on exclusion for all full texts (n=296)

	Study name	Author	Year	Journal	Reason for
1	Physical activity and quality of life among college students without comorbidities for cardiometabolic diseases: systematic review and meta-analysis	Abrantes et al.	2022	Qual Life Res	Not 100% RCT
2	Effect of hip abductors and lateral rotators' muscle strengthening on pain and functional outcome in adult patients with patellofemoral pain: A systematic review and meta-analysis	Alammari et al.	2023	Journal of Back & Musculoskeletal Rehabilitation	Not 100% RCT
3	The Role of Physical Exercise in the Prevention of Musculoskeletal Disorders in Manual Workers: A Systematic Review and Meta-Analysis	Bullo et al.	2024	Med Lav	Not 100% RCT
4	Effects of supervised exercise on aerobic capacity and quality of life in patients with chronic liver disease and patients who underwent liver transplantation: a systematic review and meta-analysis	Choo et al.	2022	Int J Rehabil Res	Not 100% RCT
5	Effects of High-Intensity Interval Training (HIIT) on Patients with Musculoskeletal Disorders: A Systematic Review and Meta-Analysis with a Meta-Regression and Mapping Report	Cuenca-Martinez et al.	2022	Diagnostics (Basel)	Not 100% RCT
6	Aerobic Physical Exercise for Pain Intensity, Aerobic Capacity, and Quality of Life in Patients With Chronic Pain: A Systematic Review and Meta-Analysis	Garcia-Correa et al.	2021	J Phys Act Health	Not 100% RCT
7	Effect of exercise on pain processing and motor output in people with knee osteoarthritis: a systematic review and meta-analysis	Hall et al.	2020	Osteoarthritis Cartilage	Not 100% RCT
8	The Effects of Workplace Physical Activity Programs on Musculoskeletal Pain: A Systematic Review and Meta-Analysis	Moreira-Silva et al.	2016	Workplace Health Saf	Not 100% RCT
9	Aerobic, resistance and combined exercise training for patients with amyotrophic lateral sclerosis: a systematic review and meta-analysis	Rahmati et al.	2021	Physiotherapy	Not 100% RCT
10	Effects of Resistance Training on Pain Control and Physical Function in Older Adults With Low Back Pain: A Systematic Review With Meta-analysis	Syroyid et al.	2023	Journal of Geriatric Physical Therapy	Not 100% RCT
11	Effect of various exercise protocols on neuropathic pain in individuals with type 2 diabetes with peripheral neuropathy: A systematic review and meta-analysis	Tatikola et al.	2022	Diabetes Metab Syndr	Not 100% RCT
12	Efficacy of Aerobic Exercise for Treatment of Chronic Low Back Pain	Xian-Guo et al.	2015	American Journal of Physical Medicine & Rehabilitation	Not 100% RCT
13	A Systematic Review and Meta-Analysis of Resistance Training on Quality of Life, Depression, Muscle Strength, and Functional Exercise Capacity in Older Adults Aged 60 Years or More	Khodadad et al.	2023	Biol Res Nurs	Not 100% RCT
14	The effect of aerobic exercise on the number of migraine days, duration and pain intensity in migraine: a systematic literature review and meta-analysis	Lemmens et al.	2019	J Headache Pain	Not 100% RCT
15	Effectiveness of aquatic exercise for musculoskeletal conditions: a meta-analysis	Barker et al.	2014	Arch Phys Med Rehabil	Not 100% RCT
16	The Effect of Exercise for the Prevention and Treatment of Cancer-Related Lymphedema: A Systematic Review with Meta-analysis	Hayes et al.	2022	Med Sci Sports Exerc	Not 100% RCT
17	Efficacy of Aerobic Exercises For Osteoarthritis (part II): A Meta-analysis	Brosseau et al.	2004	Physical Therapy Reviews	Not 100% RCT
18	Complementary and alternative exercise for fibromyalgia: a meta-analysis	Mist et al.	2013	J Pain Res	Not 100% RCT
19	Walking exercise for chronic musculoskeletal pain: systematic review and meta-analysis	O'Connor et al.	2015	Arch Phys Med Rehabil	Not 100% RCT
20	Effects of Resistance Training on Pain Control and Physical Function in Older Adults With Low Back Pain: A Systematic Review With Meta-analysis	Syroyid et al.	2022	J Geriatr Phys Ther	Not 100% RCT
21	Efficacy of home-based physical activity interventions in patients with autoimmune rheumatic diseases: A systematic review and meta-analysis	Sieczkowska et al.	2021	Semin Arthritis Rheum	Not 100% RCT
22	Effects of high-intensity training on the quality of life of cancer patients and survivors: a systematic review with meta-analysis	Lavín-Pérez et al.	2021	Sci Rep	Not 100% RCT
23	The effect of exercise on aromatase inhibitor-induced musculoskeletal symptoms in breast cancer survivors :a systematic review and meta-analysis	Lu et al.	2020	Support Care Cancer	Not 100% RCT
24	Tai Chi and Qigong for cancer-related symptoms and quality of life: a systematic review and meta-analysis	Wayne et al.	2018	J Cancer Surviv	Not 100% RCT

25	The effects of exercise on decreasing pain and increasing function in patients with patellofemoral pain syndrome: a systematic review	Frye et al.	2012	Sports Health	Not 100% RCT
26	Effects of yoga on patients with chronic nonspecific neck pain: A PRISMA systematic review and meta-analysis	Li et al.	2019	Medicine (Baltimore)	Not 100% RCT
27	Exercise-induced changes in central sensitization outcomes in individuals with chronic musculoskeletal pain: A systematic review with meta-analysis	Chen et al.	2024	Eur J Pain	Not 100% RCT
28	A Systematic Review and Meta-Analysis of Mindfulness-Based (Baduanjin) Exercise for Alleviating Musculoskeletal Pain and Improving Sleep Quality in People with Chronic Diseases	Zou et al.	2018	Int J Environ Res Public Health	Not 100% RCT
29	Effects of exercise on physical outcomes of breast cancer survivors receiving hormone therapy - A systematic review and meta-analysis	Boing et al.	2020	Maturitas	Not 100% RCT
30	Creative versus repetitive dance therapies to reduce the impact of fibromyalgia and pain: A systematic review and meta-analysis	Murillo-Garcia et al.	2022	Complement Ther Clin Pract	Not 100% RCT
31	Effectiveness of exercise versus normal activity on acute low back pain: an integrative synthesis and meta-analysis	McLain et al.	1999	Online J Knowl Synth Nurs	Not 100% RCT
32	Exercise in the Aquatic Environment for People With Primary Hip Osteoarthritis: A Systematic Review and Meta-analyses	Geigle et al.	2022	Journal of Aquatic Physical Therapy	Not 100% RCT
33	Effect of a telehealth-based exercise intervention on the physical activity of patients with breast cancer: A systematic review and meta-analysis	Peng et al.	2022	Asia Pac J Oncol Nurs	Not 100% RCT
34	Exercise, especially combined stretching and strengthening exercise, reduces myofascial pain: a systematic review	Diz et al.	2017	J Physiother	Not 100% RCT
35	Use of Wearable Activity Trackers to Improve Physical Activity Behavior in Patients With Rheumatic and Musculoskeletal Diseases: A Systematic Review and Meta-Analysis	Davergne et al.	2019	Arthritis Care Res (Hoboken)	Not 100% RCT
36	Resistance, Motor Control, and Mindfulness-Based Exercises Are Effective for Treating Chronic Nonspecific Neck Pain: A Systematic Review With Meta-Analysis and Dose-Response Meta-Regression	Mueller et al.	2023	J Orthop Sports Phys Ther	Not 100% RCT
37	Effects of resistance training on the health-related quality of life of patients with rheumatic diseases: Systematic review with meta-analysis and meta-regression	Sieczkowska et al.	2020	Semin Arthritis Rheum	Not 100% RCT
38	Exercise-based rehabilitation on functionality and quality of life in head and neck cancer survivors. A systematic review and meta-analysis	Perez et al.	2023	Scientific reports	Not 100% RCT
39	Exercise training for adults undergoing maintenance dialysis	Bernier-Jean et al.	2022	Cochrane Database Syst Rev	Not 100% RCT
40	Effects of exercise on neuropathic pain in cancer survivors: a systematic review and meta-analysis	Khemthong et al.	2024	Oncology Nursing Forum	Not 100% RCT
41	Effects of exercise-based interventions on gluteal tendinopathy. Systematic review with meta-analysis	Patricio	2024	Sci Rep	Not 100% RCT
42	Tai chi for rheumatoid arthritis	Mudano et al.	2019	Cochrane Database of Systematic Reviews	Not 100% RCT
43	Effects of home-based exercise intervention on health-related quality of life for patients with ankylosing spondylitis: a meta-analysis	Liang et al.	2015	Clin Rheumatol	Not 100% RCT
44	Effects of traditional Chinese exercise on physiological indicators and quality of life in patients with coronary heart disease: A systematic review and meta-analysis	Wang et al.	2023	Medicine (Baltimore)	Not 100% RCT
45	Pain sensitivity is reduced by exercise training: Evidence from a systematic review and meta-analysis	Belavy et al.	2021	Neurosci Biobehav Rev	Not 100% RCT
46	Effects of Conventional Exercises on Lower Back Pain and/or Pelvic Girdle Pain in Pregnancy: A Systematic Review and Meta-Analysis	Kandru et al.	2023	Cureus	Not English language
47	Effects of physical exercise at the workplace for treatment of low back pain: a systematic review with meta-analysis	Maciel et al.	2018	Rev Bras Med Trab	Not English language
48	抗阻运动在类风湿关节炎病人中应用效果的系统评价	冷雨飞 et al.	2020	Chinese Nursing Research	Not English language
49	运动疗法对提高干细胞移植病人生命 质量的Meta分析	方雪 et al.	2019	Chinese Nursing Research	Not English language
50	[Effect of functional exercises on patients with rheumatoid arthritis: a meta-analysis]	Wang et al.	2018	Beijing Da Xue Xue Bao Yi Xue Ban	Not English language
51	Effect of pilates training on pain and disability in patients with chronic low back pain: a systematic review and meta-analysis based on randomized controlled trials	Huang et al.	2023	Phys Act Nutr	Wrong comparator
52	Yoga for treating low back pain: a systematic review and meta-analysis	Anheyer et al.	2022	Pain	Wrong comparator

53	Effectiveness of exercises by telerehabilitation on pain, physical function and quality of life in people with physical disabilities: a systematic review of randomised controlled trials with GRADE recommendations	Dias et al.	2021	Br J Sports Med	Wrong comparator
54	Is aquatic exercise more effective than land-based exercise for knee osteoarthritis?	Dong et al.	2018	Medicine (Baltimore)	Wrong comparator
55	Exercise therapy for chronic fatigue syndrome	Edmonds et al.	2015	Cochrane Database of Systematic Reviews	Wrong comparator
56	Providing exercise instructions using multimedia may improve adherence but not patient outcomes: a systematic review and meta-analysis	Emmerson et al.	2019	Clin Rehabil	Wrong comparator
57	Prescription of exercises for the treatment of chronic pain along the continuum of nociplastic pain: A systematic review with meta-analysis	Ferro et al.	2021	Eur J Pain	Wrong comparator
58	Systematic review: strategies for using exercise therapy to improve outcomes in chronic low back pain	Hayden et al.	2005	Ann Intern Med	Wrong comparator
59	Comparing Telerehabilitation and Home-based Exercise for Shoulder Disorders: A Systematic Review and Meta-analysis	Huang et al.	2024	Arch Phys Med Rehabil	Wrong comparator
60	Exercise compared to a control condition or other conservative treatment options in patients with Greater Trochanteric Pain Syndrome: a systematic review and meta-analysis of randomized controlled trials	Kjeldsen et al.	2024	Physiotherapy	Wrong comparator
61	Exercise therapy for chronic fatigue syndrome	Larun et al.	2016	Cochrane Database of Systematic Reviews	Wrong comparator
62	Baduanjin exercise for low back pain: A systematic review and meta-analysis	Li et al.	2019	Complement Ther Med	Wrong comparator
63	Therapeutic Effects of Traditional Chinese Exercises on Musculoskeletal Pain: A Systematic Review and Meta-Analysis	Li et al.	2021	Pain Res Manag	Wrong comparator
64	Supervised Training Compared With No Training or Self-training in Patients With Subacromial Pain Syndrome: A Systematic Review and Meta-analysis	Liaghat et al.	2021	Arch Phys Med Rehabil	Wrong comparator
65	Effectiveness of aquatic exercise for treatment of knee osteoarthritis: Systematic review and meta-analysis	Lu et al.	2015	Z Rheumatol	Wrong comparator
66	High Intensity Exercise for Walking Competency in Individuals with Stroke: A Systematic Review and Meta-Analysis	Luo et al.	2019	J Stroke Cerebrovasc Dis	Wrong comparator
67	Pilates for neck pain: A systematic review and meta-analysis of randomised controlled trials	Martini et al.	2022	J Bodyw Mov Ther	Wrong comparator
68	Effectiveness of walking versus mind-body therapies in chronic low back pain: A systematic review and meta-analysis of recent randomized controlled trials	Nduwimana et al.	2020	Medicine (Baltimore)	Wrong comparator
69	The effectiveness of blood-flow restricted resistance training in the musculoskeletal rehabilitation of patients with lower limb disorders: A systematic review and meta-analysis	Nitzsche et al.	2021	Clin Rehabil	Wrong comparator
70	Region-specific Exercises vs General Exercises in the Management of Spinal and Peripheral Musculoskeletal Disorders: A Systematic Review With Meta-analyses of Randomized Controlled Trials	Ouellet et al.	2021	Arch Phys Med Rehabil	Wrong comparator
71	Tai chi chuan exercise for patients with breast cancer: a systematic review and meta-analysis	Pan et al.	2015	Evid Based Complement Alternat Med	Wrong comparator
72	Effects of Workplace-Based Intervention for Shoulder Pain: A Systematic Review and Meta-analysis	Picon et al.	2021	Journal of Occupational Rehabilitation	Wrong comparator
73	Walking, Cycling, and Swimming for Nonspecific Low Back Pain: A Systematic Review With Meta-analysis	Pocovi et al.	2022	The Journal of orthopaedic and sports physical therapy	Wrong comparator
74	The effectiveness of home-based therapeutic exercises on adults with fibromyalgia: a systematic review and meta-analysis	Saleh et al.	2023	Int J Rehabil Res	Wrong comparator
75	Is multi-joint or single joint strengthening more effective in reducing pain and improving function in women with patellofemoral pain syndrome? A systematic review and meta-analysis	Scali et al.	2018	Int J Sports Phys Ther	Wrong comparator
76	The Effect of Hydrokinetic Therapy on Patients with Low Back Pain: A Systematic Review and Meta-Analysis	Serra et al.	2023	Muscles, Ligaments & Tendons Journal (MLTJ)	Wrong comparator
77	Aquatic Exercises in the Treatment of Low Back Pain: A Systematic Review of the Literature and Meta-Analysis of Eight Studies	Shi et al.	2018	Am J Phys Med Rehabil	Wrong comparator
78	Effectiveness of home-based exercise interventions on pain, physical function and quality of life in individuals with knee osteoarthritis: a systematic review and meta-analysis	Si et al.	2023	J Orthop Surg Res	Wrong comparator
79	The effects of walking intervention in patients with chronic low back pain: A meta-analysis of randomized controlled trials	Sitthipornvorakul et al.	2018	Musculoskelet Sci Pract	Wrong comparator

80	Should exercises be painful in the management of chronic musculoskeletal pain? A systematic review and meta-analysis	Smith et al.	2017	Br J Sports Med	Wrong comparator
81	Effectiveness of Therapeutic Exercise in Fibromyalgia Syndrome: A Systematic Review and Meta-Analysis of Randomized Clinical Trials	Sosa-Reina et al.	2017	Biomed Res Int	Wrong comparator
82	Quadriceps strengthening with blood flow restriction for the rehabilitation of patients with knee conditions: A systematic review with meta-analysis	Van Cant et al.	2020	Journal of Back & Musculoskeletal Rehabilitation	Wrong comparator
83	The effectiveness of walking versus exercise on pain and function in chronic low back pain: a systematic review and meta-analysis of randomized trials	Vanti et al.	2019	Disabil Rehabil	Wrong comparator
84	A meta-analysis of core stability exercise versus general exercise for chronic low back pain	Wang et al.	2012	PLoS One	Wrong comparator
85	Efficacy and safety of aquatic exercise in knee osteoarthritis: A systematic review and meta-analysis of randomized controlled trials	Xu et al.	2023	Clin Rehabil	Wrong comparator
86	Home-based vs center-based exercise on patient-reported and performance-based outcomes for knee osteoarthritis: a systematic review with meta-analysis	Zhang et al.	2024	Frontiers in public health	Wrong comparator
87	Effects of Exergaming on Musculoskeletal Pain in Older Adults: Systematic Review and Meta-analysis	Mo et al.	2023	JMIR Serious Games	Wrong comparator
88	Effects of supervised exercise compared to non-supervised exercise early after total hip replacement on patient-reported function, pain, health-related quality of life and performance-based function - a systematic review and meta-analysis of randomized controlled trials	Hansen et al.	2019	Clin Rehabil	Wrong comparator
89	Pool-based exercise for amelioration of pain in adults with fibromyalgia syndrome: A systematic review and meta-analysis	Galvao-Moreira et al.	2021	Mod Rheumatol	Wrong comparator
90	Effects of traditional Chinese exercise therapy on pain scores, sleep quality, and anxiety-depression symptoms in fibromyalgia patients: a systematic review and meta-analysis	Wang et al.	2024	BMC Musculoskelet Disord	Wrong comparator
91	Traditional Chinese Mind and Body Exercises for Neck Pain: A Meta-Analysis of Randomized Controlled Trials	Xie et al.	2021	Pain Research & Management	Wrong comparator
92	The Effects of Physical Exercise on Pain Management in Patients with Knee Osteoarthritis: A Systematic Review with Metanalysis	Rocha et al.	2020	Rev Bras Ortop (Sao Paulo)	Wrong comparator
93	Water-Based Exercise on Functioning and Quality of Life in Poststroke Persons: A Systematic Review and Meta-Analysis	Saquetto et al.	2019	J Stroke Cerebrovasc Dis	Wrong comparator
94	Efficacy of Pilates on Pain, Functional Disorders and Quality of Life in Patients with Chronic Low Back Pain: A Systematic Review and Meta-Analysis	Yu et al.	2023	Int J Environ Res Public Health	Wrong comparator
95	Efficacy of exercise for improving functional outcomes for patients undergoing total hip arthroplasty: A meta-analysis	Wu et al.	2019	Medicine	Wrong comparator
96	Yoga for functional ability, pain and psychosocial outcomes in musculoskeletal conditions: a systematic review and meta-analysis	Ward et al.	2013	Musculoskeletal Care	Wrong comparator
97	Effects of physical exercise on muscle function of the knee, pain and quality of life in postmenopausal women with knee osteoarthritis: A systematic review with meta-analysis	Wolf et al.	2024	Musculoskeletal science & practice	Wrong comparator
98	Yoga for Treating Headaches: a Systematic Review and Meta-analysis	Anheyer et al.	2020	J Gen Intern Med	Wrong comparator
99	Strength Training vs. Aerobic Training for Managing Pain and Physical Function in Patients with Knee Osteoarthritis: A Systematic Review and Meta-Analysis	Ceballos-Laita et al.	2023	Healthcare (Basel)	Wrong comparator
100	Posterior-Chain Resistance Training Compared to General Exercise and Walking Programmes for the Treatment of Chronic Low Back Pain in the General Population: A Systematic Review and Meta-Analysis	Tataryn et al.	2021	Sports Medicine - Open	Wrong comparator
101	The effects of Pilates exercise in comparison to other forms of exercise on pain and disability in individuals with chronic non-specific low back pain: A systematic review with meta-analysis	Wong et al.	2023	Musculoskeletal Care	Wrong comparator
102	Exercise Intervention for Chronic Pain Management, Muscle Strengthening, and Functional Score in Obese Patients with Chronic Musculoskeletal Pain: A Systematic Review and Meta-analysis	Tamin et al.	2018	Acta Med Indones	Wrong comparator
103	Effectiveness of pharmacological and non-pharmacological therapy on pain intensity and disability in older people with chronic nonspecific low back pain: a systematic review with meta-analysis	Soares	2023	Eur Spine J	Wrong comparator
104	A systematic review and meta-analysis of yoga for low back pain	Cramer et al.	2013	Clin J Pain	Wrong comparator

105	The effect of aerobic exercise on the number of migraine days, duration and pain intensity in migraine: a systematic literature review and meta-analysis: Official Journal of the Italian Society for the Study of Headaches	Lemmens et al.	2019	The journal of headache and pain	Wrong comparator
106	Aquatic therapy improves self-reported sleep quality in fibromyalgia patients: a systematic review and meta-analysis	Bravo et al.	2024	Sleep Breath	Wrong comparator
107	Workplace-Based Interventions for Neck Pain in Office Workers: Systematic Review and Meta-Analysis	Xiaoqi et al.	2018	Physical Therapy	Wrong comparator
108	The efficacy of exercise therapy for rotator cuff related shoulder pain according to the FITT principle: a systematic review with meta-analyses	Lafrance et al.	2024	The Journal of orthopaedic and sports physical therapy	Wrong intervention
109	Core Stabilization Exercise in Prenatal and Postnatal Women With Urinary Incontinence: A Systematic Review and Meta-analysis of Randomized Controlled Trials	Chin-Yin et al.	2023	American Journal of Physical Medicine & Rehabilitation	Wrong intervention
110	Pain Science Education Plus Exercise Therapy in Chronic Nonspecific Spinal Pain: A Systematic Review and Meta-analyses of Randomized Clinical Trials	Bonatesta et al.	2022	J Pain	Wrong intervention
111	Aquatic exercise training for fibromyalgia	Bidonde et al.	2014	Cochrane Database Syst Rev	Wrong intervention
112	Effects of lifestyle physical activity and sedentary behaviour interventions on disease activity and patient- and clinician- important health outcomes in rheumatoid arthritis: a systematic review with meta-analysis	Brady et al.	2023	BMC Rheumatol	Wrong intervention
113	Effectiveness of aquatic training based on aerobic and strengthening exercises in patients with fibromyalgia: systematic review with meta-analysis	Correyero-Leon et al.	2024	Explore (NY)	Wrong intervention
114	Exercise as effective as surgery in improving quality of life, disability, and pain for large to massive rotator cuff tears: A systematic review & meta-analysis	Fahy et al.	2022	Musculoskelet Sci Pract	Wrong intervention
115	Systematic Review and Meta-Analysis Suggest Strength Training and Workplace Modifications May Reduce Neck Pain in Office Workers	Frutiger et al.	2021	Pain Pract	Wrong intervention
116	The effect of Pilates exercise training for scoliosis on improving spinal deformity and quality of life: Meta-analysis of randomized controlled trials	Gou et al.	2021	Medicine (Baltimore)	Wrong intervention
117	Effect of exercise in the treatment of chronic low back pain: a systematic review, emphasising type and dose of exercise	Hilde et al.	1998	Physical Therapy Reviews	Wrong intervention
118	The effect of pelvic floor muscle-strengthening exercises on low back pain: a systematic review and meta-analysis on randomized clinical trials	Kazeminia et al.	2023	Neurol Sci	Wrong intervention
119	Multimodal Interventions Including Rehabilitation Exercise for Older Adults With Chronic Musculoskeletal Pain: A Systematic Review and Meta-analyses of Randomized Controlled Trials	Kechichian et al.	2022	J Geriatr Phys Ther	Wrong intervention
120	Effectiveness of Mobile Health-Based Exercise Interventions for Patients with Peripheral Artery Disease: Systematic Review and Meta-Analysis	Kim et al.	2021	JMIR Mhealth Uhealth	Wrong intervention
121	Effects of eccentric exercise in patients with subacromial impingement syndrome: a systematic review and meta-analysis	Larsson et al.	2019	BMC Musculoskelet Disord	Wrong intervention
122	Efficacy and Safety of Lower Limb Progressive Resistance Exercise for Patients With Total Knee Arthroplasty: A Meta-analysis of Randomized Controlled Trials	Liu et al.	2021	Arch Phys Med Rehabil	Wrong intervention
123	A Systematic Review and Meta-Analysis on the Effectiveness of Graded Activity and Graded Exposure for Chronic Nonspecific Low Back Pain	Lopez-de-Uralde-Villanueva et al.	2016	Pain Med	Wrong intervention
124	Knee osteoarthritis pain and stretching exercises: a systematic review and meta-analysis	Luan et al.	2022	Physiotherapy	Wrong intervention
125	The effects of resistance training on muscle strength, joint pain, and hand function in individuals with hand osteoarthritis: a systematic review and meta-analysis	Magni et al.	2017	Arthritis Res Ther	Wrong intervention
126	Physical activity for primary dysmenorrhea: aÂ systematic review and meta-analysis of randomized controlled trials	Matthewman et al.	2018	Am J Obstet Gynecol	Wrong intervention
127	Efficacy of heavy eccentric calf training for treating mid-portion Achilles tendinopathy: a systematic review and meta-analysis	Murphy et al.	2019	Br J Sports Med	Wrong intervention
128	Effectiveness of Neuromuscular exercises (NEMEX) in knee osteoarthritis: A Systematic Review with meta-analysis	Sabharwal et al.	2022	Advances in Rehabilitation	Wrong intervention
129	Physical conditioning as part of a return to work strategy to reduce sickness absence for workers with back pain	Schaafsma et al.	2013	Cochrane Database of Systematic Reviews	Wrong intervention

130	Exercise interventions for the treatment of chronic low back pain: a systematic review and meta-analysis of randomised controlled trials	Searle et al.	2015	Clin Rehabil	Wrong intervention
131	Specific or general exercise strategy for subacromial impingement syndrome-does it matter? A systematic literature review and meta analysis	Shire et al.	2017	BMC Musculoskelet Disord	Wrong intervention
132	Trunk-strengthening exercises for chronic low back pain: a systematic review	Slade et al.	2006	J Manipulative Physiol Ther	Wrong intervention
133	Effects of yoga on chronic neck pain: a systematic review and meta-analysis	Cramer et al.	2017	Clin Rehabil	Wrong intervention
134	The Value of Preoperative Exercise and Education for Patients Undergoing Total Hip and Knee Arthroplasty: A Systematic Review and Meta-Analysis	Moyer et al.	2017	JBJS Rev	Wrong intervention
135	Individualized Exercise in Chronic Non-Specific Low Back Pain: A Systematic Review with Meta-Analysis on the Effects of Exercise Alone or in Combination with Psychological Interventions on Pain and Disability	Fleckenstein et al.	2022	J Pain	Wrong intervention
136	Effect of Physical Exercise Programs on Myofascial Trigger Points-Related Dysfunctions: A Systematic Review and Meta-analysis	Guzmán-Pavón et al.	2020	Pain Med	Wrong intervention
137	Can we explain heterogeneity among randomized clinical trials of exercise for chronic back pain? A meta-regression analysis of randomized controlled trials	Ferreira et al.	2010	Physical Therapy	Wrong intervention
138	Exercise therapy for chronic low back pain	Hayden et al.	2021	Cochrane Database Syst Rev	Wrong intervention
139	Exercise interventions in lateral elbow tendinopathy have better outcomes than passive interventions, but the effects are small: a systematic review and meta-analysis of 2123 subjects in 30 trials	Karanasios et al.	2021	Br J Sports Med	Wrong intervention
140	How do the target concepts of pain science education combined with exercise contribute to the effect on pain intensity and disability in patients with chronic spinal pain? A systematic review and meta-analysis with moderator analysis	Núñez-Cortés et al.	2024	Neurosci Biobehav Rev	Wrong intervention
141	The effects of exercise on function and pain following total hip arthroplasty: a systematic literature review and meta-analysis	Tang et al.	2022	Physical Therapy Reviews	Wrong intervention
142	The influence of dosing on effect size of exercise therapy for musculoskeletal foot and ankle disorders: a systematic review	Young et al.	2018	Braz J Phys Ther	Wrong intervention
143	An update of stabilisation exercises for low back pain: a systematic review with meta-analysis	Smith et al.	2014	BMC Musculoskelet Disord	Wrong intervention
144	Physical exercise improved muscle strength and pain on neck and shoulder in military pilots	Heng et al.	2022	Front Physiol	Wrong intervention
145	Are mind-body therapies effective for relieving cancer-related pain in adults? A systematic review and meta-analysis	Danon et al.	2022	Psycho-Oncology	Wrong intervention
146	The addition of blood flow restriction to resistance exercise in individuals with knee pain: a systematic review and meta-analysis	Cuyul-Vásquezet al.	2020	Braz J Phys Ther	Wrong intervention
147	Exercise Interventions for Persistent Non-Specific Low Back Pain - Does Matching Outcomes to Treatment Targets Make a Difference? A Systematic Review and Meta-Analysis	Wood et al.	2021	J Pain	Wrong intervention
148	Whole Body Vibration Exercise for Chronic Musculoskeletal Pain: A Systematic Review and Meta-analysis of Randomized Controlled Trials	Dong et al.	2019	Arch Phys Med Rehabil	Wrong intervention
149	Is it necessary to perform prehabilitation exercise for patients undergoing total knee arthroplasty: meta-analysis of randomized controlled trials	Chen et al.	2018	Phys Sportsmed	Wrong intervention
150	Effects of Low Back Pain Exercises on Pain Symptoms and Activities of Daily Living: A Systematic Review and Meta-Analysis	Zhang et al.	2022	Perceptual & Motor Skills	Wrong intervention
151	Effect of Trunk-Focused Exercises on Pain, Disability, Quality of Life, and Trunk Physical Fitness in Low Back Pain and How Potential Effect Modifiers Modulate Their Effects: A Systematic Review With Meta-analyses	Prat-Luri et al.	2023	J Orthop Sports Phys Ther	Wrong intervention
152	Advice to Stay Active or Structured Exercise in the Management of Sciatica: A Systematic Review and Meta-analysis	Fernandez et al.	2015	Spine (Phila Pa 1976)	Wrong intervention
153	The Effectiveness of Exercise-Based Rehabilitation in People With Hand Osteoarthritis: A Systematic Review With Meta-analysis	Huang et al.	2024	The Journal of orthopaedic and sports physical therapy	Wrong intervention
154	The effectiveness of hip interventions in patients with low-back pain: A systematic review and meta-analysis	Ceballos-Laita et al.	2023	Braz J Phys Ther	Wrong intervention
155	Exercises for mechanical neck disorders: A Cochrane review update	Gross et al.	2016	Man Ther	Wrong intervention

156	Effects of Sling Exercise for Neck Pain: A Systematic Review and Meta-Analysis	Lin et al.	2021	PTJ: Physical Therapy & Rehabilitation Journal	Wrong intervention
157	The effect of exercise post vertebral augmentation in osteoporotic patients: A systematic review and meta-analysis	Than et al.	2023	J Orthop Res	Wrong intervention
158	Effect of Low-Intensity Bloodflow Restriction Training on Nontraumatic Knee Joint Conditions: A Systematic Review and Meta-analysis	Peng et al.	2024	Sports Health	Wrong intervention
159	Virtual reality training versus conventional rehabilitation for chronic neck pain: A systematic review and meta-analysis	Hao et al.	2024	Pm r	Wrong intervention
160	Exercise Therapy Is Effective for Improvement in Range of Motion, Function, and Pain in Patients With Frozen Shoulder: A Systematic Review and Meta-analysis	Mertens et al.	2022	Arch Phys Med Rehabil	Wrong intervention
161	Effects of Blood Flow Restriction Training on Muscle Strength and Pain in Patients With Knee Injuries: A Meta-Analysis	Li et al.	2021	Am J Phys Med Rehabil	Wrong intervention
162	Core training for pain management and functional improvement in patients with patellofemoral pain syndrome: a systematic review and meta-analysis	Wang et al.	2024	Am J Phys Med Rehabil	Wrong intervention
163	Effectiveness of balance training on pain and functional outcomes in knee osteoarthritis: A systematic review and meta-analysis	Prabhakar et al.	2022	F1000Res	Wrong intervention
164	The effectiveness of a combined exercise and psychological treatment programme on measures of nervous system sensitisation in adults with chronic musculoskeletal pain - a systematic review and meta-analysis	Deegan et al.	2024	BMC Musculoskelet Disord	Wrong intervention
165	Medical exercise therapy alone versus arthroscopic partial meniscectomy followed by medical exercise therapy for degenerative meniscal tear: a systematic review and meta-analysis of randomized controlled trials	Ma et al.	2020	J Orthop Surg Res	Wrong intervention
166	Is Exercise Rehabilitation an Effective Adjuvant to Clinical Treatment for Myofascial Trigger Points? A Systematic Review and Meta-Analysis	Zhou et al.	2023	J Pain Res	Wrong intervention
167	Isokinetic muscle strengthening for knee osteoarthritis: A systematic review of randomized controlled trials with meta-analysis	Coudeyre et al.	2016	Ann Phys Rehabil Med	Wrong intervention
168	Effectiveness of mHealth interventions to improve pain intensity and functional disability in patients with hip or knee osteoarthritis: a systematic review and meta-analysis	Mapinduzi et al.	2024	Arch Phys Med Rehabil	Wrong intervention
169	Effects of Exercise Intervention (with and without Joint Mobilization) in Patients with Adhesive Capsulitis: A Systematic Review and Meta-Analysis	Lee et al.	2023	Healthcare (Basel)	Wrong intervention
170	Effectiveness of exercise in office workers with neck pain: A systematic review and meta-analysis	Louw et al.	2017	S Afr J Physiother	Wrong intervention
171	Exercise therapy for treatment of non-specific low back pain	Hayden et al.	2005	Cochrane Database of Systematic Reviews	Wrong intervention
172	Effectiveness of exercise programs to reduce low back pain among nurses and nursing assistants: A systematic review and meta-analysis	Indrayani et al.	2024	Journal of safety research	Wrong intervention
173	Sling exercise for chronic low back pain: a systematic review and meta-analysis	Yue et al.	2014	PLoS One	Wrong intervention
174	The Impact of a Stabilization Exercise on Neck Pain: A Systematic Review and Meta-analysis	Wu et al.	2020	J Neurol Surg A Cent Eur Neurosurg	Wrong intervention
175	The effect of exercise on stabilizing and strengthening core muscles for patients with symptomatic herniated lumbar disc: A systematic review and meta-analysis	Choo et al.	2024	Asian J Surg	Wrong intervention
176	Exercises for prevention of recurrences of low-back pain	Choi et al.	2010	Cochrane Database of Systematic Reviews	Wrong intervention
177	Prevention of Low Back Pain: A Systematic Review and Meta-analysis	Steffens et al.	2016	JAMA Intern Med	Wrong intervention
178	Exercise programs may be effective in preventing a new episode of neck pain: a systematic review and meta-analysis	de Campos et al.	2018	J Physiother	Wrong intervention
179	Efficacy of workplace interventions for shoulder pain: a systematic review and meta-analysis	Lowry et al.	2017	Journal of Rehabilitation Medicine (Stiftelsen Rehabiliteringsinformation)	Wrong intervention
180	Therapeutic exercise for people with osteoarthritis of the hip or knee. A systematic review	Fransen et al.	2002	J Rheumatol	Wrong intervention
181	Exercise therapy for whiplash-associated disorders: a systematic review and meta-analysis	Chrcanovic et al.	2022	Scandinavian journal of pain	Wrong intervention

182	Exercise-Based Interventions Are Effective in the Management of Patients with Thumb Carpometacarpal Osteoarthritis: A Systematic Review and Meta-Analysis of Randomised Controlled Trials	Karanasios et al.	2024	Healthcare (Basel)	Wrong intervention
183	The Beneficial Effects of Eccentric Exercise in the Management of Lateral Elbow Tendinopathy: A Systematic Review and Meta-Analysis	Yoon et al.	2021	J Clin Med	Wrong intervention
184	Complementary and integrative health approaches to manage chronic pain in U.S. military populations: Results from a systematic review and meta-analysis, 1985-2019	Donahue et al.	2021	Psychol Serv	Wrong intervention
185	Dose-response effect of lower limb resistance training volume on pain and function of women with patellofemoral pain: A systematic review and meta-regression	de Oliveira et al.	2023	Phys Ther Sport	Wrong intervention
186	Effectiveness of movement control exercise on patients with non-specific low back pain and movement control impairment: A systematic review and meta-analysis	Luomajoki et al.	2018	Musculoskelet Sci Pract	Wrong intervention
187	Effect of total-body prehabilitation on postoperative outcomes: a systematic review and meta-analysis	Mina et al.	2014	Physiotherapy	Wrong intervention
188	Rehabilitation after lumbar spine surgery in adults: a systematic review with meta-analysis	Manni et al.	2023	Arch Physiother	Wrong intervention
189	Rehabilitation after lumbar disc surgery: an update Cochrane review	Ostelo et al.	2009	Spine (Phila Pa 1976)	Wrong intervention
190	The Effect of Exercise Interventions After Lumbar Decompression Surgery: A Systematic Review and Meta-Analysis	Ã–zden et al.	2022	World Neurosurg	Wrong intervention
191	Meta-analysis: exercise therapy for nonspecific low back pain	Hayden et al.	2005	Ann Intern Med	Wrong intervention
192	Effect of lower limb resistance training on ICF components in chronic stroke: A systematic review and meta-analysis of RCTs	Pereira et al.	2023	Ann Phys Rehabil Med	Wrong intervention
193	Effectiveness of Exercise Interventions for Preventing Neck Pain: A Systematic Review With Meta-analysis of Randomized Controlled Trials	Teichert et al.	2023	J Orthop Sports Phys Ther	Wrong intervention
194	The optimal dose of pain neuroscience education added to an exercise programme for patients with chronic spinal pain: a systematic review and dose-response meta-analysis	Núñez-Cortés et al.	2024	Pain	Wrong intervention
195	Prevention strategies to reduce future impact of low back pain: a systematic review and meta-analysis	de Campos et al.	2021	Br J Sports Med	Wrong intervention
196	Effectiveness of Specific Exercise for Deep Cervical Muscles in Nonspecific Neck Pain: A Systematic Review and Meta-Analysis	Garzonio et al.	2022	Phys Ther	Wrong intervention
197	Effectiveness of Home-Based Exercise for Nonspecific Shoulder Pain: A Systematic Review and Meta-analysis	Liu et al.	2022	Arch Phys Med Rehabil	Wrong intervention
198	Exercise interventions for shoulder dysfunction in patients treated for head and neck cancer	Carvalho et al.	2012	Cochrane Database of Systematic Reviews	Wrong intervention
199	Effects of exercise therapy on pain and disability in patients with non-specific neck pain: A systematic review and meta-analysis	Cho et al.	2023	J Bodyw Mov Ther	Wrong intervention
200	Efficacy of strengthening or aerobic exercise on pain relief in people with knee osteoarthritis: a systematic review and meta-analysis of randomized controlled trials	Tanaka et al.	2013	Clin Rehabil	Wrong intervention
201	Addition of specific hip strengthening exercises to conventional rehabilitation therapy for low back pain: a systematic review and meta-analysis	de Jesus et al.	2020	Clin Rehabil	Wrong intervention
202	Unloaded movement facilitation exercise compared to no exercise or alternative therapy on outcomes for people with nonspecific chronic low back pain: a systematic review	Slade et al.	2007	J Manipulative Physiol Ther	Wrong intervention
203	Is adding pelvic floor muscle training to an exercise intervention more effective at improving pain in patients with non-specific low back pain? A systematic review of randomized controlled trials	Bernard et al.	2021	Physiotherapy	Wrong intervention
204	Does land-based exercise reduce pain and disability associated with hip osteoarthritis? A meta-analysis of randomized controlled trials	Fransen et al.	2010	Osteoarthritis Cartilage	Wrong intervention
205	Virtual Reality-Based Training in Chronic Low Back Pain: Systematic Review and Meta-Analysis of Randomized Controlled Trials	Li et al.	2024	J Med Internet Res	Wrong intervention
206	Does adding hip exercises to quadriceps exercises result in superior outcomes in pain, function and quality of life for people with knee osteoarthritis? A systematic review and meta-analysis	Hislop et al.	2020	Br J Sports Med	Wrong intervention
207	The effect of exercise on health-related quality of life in persons with musculoskeletal pain: A meta-analysis of randomised control trials	Amiri et al.	2022	Musculoskeletal Care	Wrong intervention
208	Effects of High-Intensity Strength Training in Adults With Knee Osteoarthritis: A Systematic Review and Meta-analysis of Randomized Controlled Trials	Hua et al.	2023	Am J Phys Med Rehabil	Wrong intervention

209	Hip and Knee Strengthening Is More Effective Than Knee Strengthening Alone for Reducing Pain and Improving Activity in Individuals With Patellofemoral Pain: A Systematic Review With Meta-analysis	Nascimento et al.	2018	J Orthop Sports Phys Ther	Wrong intervention
210	Whole-Body Vibration Exercise for Knee Osteoarthritis: A Systematic Review and Meta-Analysis	Li et al.	2015	Evidence-based Complementary & Alternative Medicine (eCAM)	Wrong intervention
211	Eccentric exercise is more effective than other exercises in the treatment of mid-portion Achilles tendinopathy: systematic review and meta-analysis	Prudencio et al.	2023	BMC Sports Sci Med Rehabil	Wrong intervention
212	The role of exercise for pain management in adults living with and beyond cancer: a systematic review and meta-analysis	Cuthbert et al.	2023	Support Care Cancer	Wrong intervention
213	Efficacy of proprioceptive training on the recovery of total joint arthroplasty patients: a meta-analysis	Zhang et al.	2020	Journal of Orthopaedic Surgery & Research	Wrong intervention
214	Hip Strengthening After Total Knee Arthroplasty: A Meta-analysis and Systematic Review	Daher et al.	2024	Arch Bone Jt Surg	Wrong intervention
215	Short-term impact of combining pain neuroscience education with exercise for chronic musculoskeletal pain: a systematic review and meta-analysis	Siddall et al.	2022	Pain	Wrong intervention
216	Graded activity and graded exposure for persistent nonspecific low back pain: a systematic review	Macedo et al.	2010	Phys Ther	Wrong intervention
217	Pelvic floor muscle training for women with lumbopelvic pain: A systematic review and meta-analysis	Vesentini et al.	2020	Eur J Pain	Wrong intervention
218	Trunk, Hip and Knee Exercise Programs for Pain Relief, Functional Performance and Muscle Strength in Patellofemoral Pain: Systematic Review and Meta-Analysis	Manojlović et al.	2021	J Pain Res	Wrong intervention
219	Exercise Therapy for Treatment of Acute Non-specific Low Back Pain: A Cochrane Systematic Review and Meta-analysis of Randomized Controlled Trials	IJzelenberg et al.	2024	Arch Phys Med Rehabil	Wrong intervention
220	The influence of exercise on pain, disability and quality of life in office workers with chronic neck pain: A systematic review and meta-analysis	Jones et al.	2024	Appl Ergon	Wrong intervention
221	Patient education improves pain and function in people with knee osteoarthritis with better effects when combined with exercise therapy: a systematic review	Goff et al.	2021	J Physiother	Wrong intervention
222	Effectiveness of therapeutic exercise for the management of cervicogenic headache: A systematic review	Becher et al.	2023	Musculoskelet Sci Pract	Wrong intervention
223	A systematic review and meta-analysis of the effect of preoperative exercise intervention on rehabilitation after total knee arthroplasty	Wang et al.	2021	Ann Palliat Med	Wrong intervention
224	The effectiveness of proprioceptive-based exercise for osteoarthritis of the knee: a systematic review and meta-analysis	Smith et al.	2012	Rheumatol Int	Wrong intervention
225	Efficacy and Safety of Blood Flow Restriction Training in Patients With Knee Osteoarthritis: A Systematic Review and Meta-Analysis	Wang et al.	2022	Arthritis Care Res (Hoboken)	Wrong intervention
226	Flexibility exercise training for adults with fibromyalgia	Kim et al.	2019	Cochrane Database of Systematic Reviews	Wrong intervention
227	Does land-based exercise-therapy improve physical activity in people with knee osteoarthritis? A systematic review with meta-analyses	Bell et al.	2022	Osteoarthritis Cartilage	Wrong outcomes
228	Effectiveness of movement and body awareness therapies in patients with fibromyalgia: a systematic review and meta-analysis	Bravo et al.	2019	Eur J Phys Rehabil Med	Wrong outcomes
229	Therapeutic Aquatic Exercise in Pregnancy: A Systematic Review and Meta-Analysis	Cancela-Carral et al.	2022	J Clin Med	Wrong outcomes
230	The Impact of Supervised Exercise Training on Traditional Cardiovascular Risk Factors in Patients With Intermittent Claudication: A Systematic Review and Meta-Analysis	Cornelis et al.	2019	Eur J Vasc Endovasc Surg	Wrong outcomes
231	The Effects of Exercise-Based Interventions on Fluid Overload Symptoms in Patients with Heart Failure: A Systematic Review and Meta-Analysis	Fu et al.	2022	Biomedicines	Wrong outcomes
232	Resistance training for rehabilitation after burn injury: A systematic literature review & meta-analysis	Gittings et al.	2018	Burns	Wrong outcomes
233	Effect of high-intensity exercise on cardiorespiratory fitness in stroke survivors: A systematic review and meta-analysis	Luo et al.	2020	Ann Phys Rehabil Med	Wrong outcomes
234	Intensive walking exercise for lower extremity peripheral arterial disease: A systematic review and meta-analysis	Lyu et al.	2016	J Diabetes	Wrong outcomes
235	The Effect of Exercise on Quality of Life, Fatigue, Physical Function, and Safety in Advanced Solid Tumor Cancers: A Meta-analysis of Randomized Control Trials	Nadler et al.	2019	J Pain Symptom Manage	Wrong outcomes

236	Effect of high-pain versus low-pain structured exercise on walking ability in people with intermittent claudication: meta-analysis	Perks et al.	2022	Br J Surg	Wrong outcomes
237	Exercise for intermittent claudication	Watson et al.	2014	Cochrane Database of Systematic Reviews	Wrong outcomes
238	The Effect of Yoga Therapy in Premenstrual Syndrome: A Systematic Review and Meta- Analysis of Randomized Controlled Trials	Ranga et al.	2024	J Obstet Gynaecol Can	Wrong outcomes
239	Pilates for breast cancer: A systematic review and meta-analysis	Espíndula et al.	2017	Rev Assoc Med Bras (1992)	Wrong outcomes
240	Evaluation of Exercise Interventions and Outcomes After Hip Arthroplasty: A Systematic Review and Meta-analysis	Saueressig et al.	2021	JAMA Netw Open	Wrong outcomes
241	Exercise for intermittent claudication	Lane et al.	2017	Cochrane Database Syst Rev	Wrong outcomes
242	Effects of the pilates method on kinesiophobia associated with chronic non-specific low back pain: Systematic review and meta-analysis	Domingues et al.	2020	J Bodyw Mov Ther	Wrong outcomes
243	Matching the Outcomes to Treatment Targets of Exercise for Low Back Pain: Does it Make a Difference? Results of Secondary Analyses From Individual Patient Data of Randomised Controlled Trials and Pooling of Results Across Trials in Comparative Meta-analysis	Wood et al.	2023	Arch Phys Med Rehabil	Wrong outcomes
244	Exercise for improving outcomes after osteoporotic vertebral fracture	Giangregorio et al.	2013	Cochrane Database of Systematic Reviews	Wrong outcomes
245	Effect of aquatic exercise on physical function and QOL in individuals with neurological disorder: A systematic review and meta-analysis	Oh et al.	2021	J Bodyw Mov Ther	Wrong outcomes
246	Effectiveness of exercise on work disability in patients with non-acute non-specific low back pain: Systematic review and meta-analysis of randomised controlled trials	Oesch et al.	2010	J Rehabil Med	Wrong outcomes
247	Supervised walking therapy in patients with intermittent claudication	Fakhry et al.	2012	J Vasc Surg	Wrong outcomes
248	Exercise for fibromyalgia: a systematic review	Busch et al.	2008	J Rheumatol	Wrong outcomes
249	Effectiveness of antenatal yoga in reducing intensity of labour pain: A systematic review and meta-analysis	Boopalan et al.	2023	Eur J Obstet Gynecol Reprod Biol X	Wrong outcomes
250	Effectiveness of dance movement therapy and dance movement interventions on cancer patients' health-related outcomes: a systematic review and meta-analysis	Abu-Odah et al.	2024	Support Care Cancer	Wrong outcomes
251	Intensity-dependent effects of exercise therapy on walking performance and aerobic fitness in symptomatic patients with lower-extremity peripheral artery disease: A systematic review and meta-analysis	Fassora et al.	2022	Vasc Med	Wrong outcomes
252	Managing fibromyalgia with complementary and alternative medical exercise: a systematic review and meta-analysis of clinical trials	Vasileios et al.	2022	Rheumatol Int	Wrong outcomes
253	The Impact of Exercise on Fatigue among Patients Undergoing Adjuvant Radiation Therapy: A Systematic Review and Meta-analysis	Halemani et al.	2022	J Caring Sci	Wrong outcomes
254	Rehabilitation to improve outcomes of lumbar fusion surgery: a systematic review with meta-analysis	Bogaert et al.	2022	Eur Spine J	Wrong population
255	The Effects of Exercise Intervention for Post-Operative Breast Cancer Patients in Korea: A Systemic Review and Meta-Analysis of Randomized Controlled Trials	Lee et al.	2021	Asian Oncology Nursing	Wrong population
256	Effect of exercise on rehabilitation of breast cancer surgery patients: A systematic review and meta-analysis of randomized controlled trials	Lin et al.	2023	Nurs Open	Wrong population
257	Effect of exercise training on heath, quality of life, exercise capacity in juvenile idiopathic arthritis: a meta-analysis of randomized controlled trials	Liu et al.	2024	Pediatric rheumatology online journal	Wrong population
258	Rehabilitation following surgery for lumbar spinal stenosis	McGregor et al.	2013	Cochrane Database Syst Rev	Wrong population
259	The Role of Exercise in the Alleviation of Neuropathic Pain Following Traumatic Spinal Cord Injuries: A Systematic Review and Meta-analysis	Toloui et al.	2023	Neurospine	Wrong population
260	Preoperative Strength Training for Clinical Outcomes Before and After Total Knee Arthroplasty: A Systematic Review and Meta-Analysis	Wu et al.	2022	Front Surg	Wrong population

261	Effect of Type and Dose of Exercise on Neuropathic Pain After Experimental Sciatic Nerve Injury: A Preclinical Systematic Review and Meta-Analysis	Matesanz-Garcia et al.	2023	J Pain	Wrong population
262	A Systematic Review and Meta-analysis of Randomized Controlled Trials of Stabilizing Exercises for Lumbopelvic Region Impact in Postpartum Women With Low Back and Pelvic Pain	Moheboleslam et al.	2022	Biol Res Nurs	Wrong population
263	The Effectiveness of Physical Exercise After Lumbar Fusion Surgery: A Systematic Review and Meta-Analysis	Ozden et al.	2022	World Neurosurg	Wrong population
264	Effects of yoga interventions on pain and pain-associated disability: a meta-analysis	Bussing et al.	2012	J Pain	Wrong study design
265	Mind-body practices for cancer-related symptoms management: an overview of systematic reviews including one hundred twenty-nine meta-analyses	Casuso-Holgado et al.	2022	Support Care Cancer	Wrong study design
266	Effects of yoga on anxiety, pain, inflammatory and stress biomarkers in patients undergoing cardiac surgery: A systematic review and meta-analysis	Chandrababu et al.	2023	Complement Ther Clin Pract	Wrong study design
267	Effectiveness of home rehabilitation exercise for patients after total knee replacement: meta analysis	Chen et al.	2018	Chinese Nursing Research	Wrong study design
268	Core Stability Exercise Versus General Exercise for Chronic Low Back Pain	Coulombe et al.	2017	J Athl Train	Wrong study design
269	Benefits of aquatic exercise in adults with and without chronic disease-A systematic review with meta-analysis	Fail et al.	2022	Scand J Med Sci Sports	Wrong study design
270	Effects of physical exercise and body weight on disease-specific outcomes of people with rheumatic and musculoskeletal diseases (RMDs): systematic reviews and meta-analyses informing the 2021 EULAR recommendations for lifestyle improvements in people with RMDs	Gwinnutt et al.	2022	RMD Open	Wrong study design
271	Dynamic exercise programs (aerobic capacity and/or muscle strength training) in patients with rheumatoid arthritis	Hurkmans et al.	2009	Cochrane Database of Systematic Reviews	Wrong study design
272	An abridged version of the Cochrane review of exercise therapy for chronic fatigue syndrome	Larun et al.	2016	Eur J Phys Rehabil Med	Wrong study design
273	The effectiveness of multidisciplinary, activity-based chronic pain interventions for adults of ethnoculturally diverse backgrounds: a systematic review with meta-analysis	Mojdehi et al.	2024	Disabil Rehabil	Wrong study design
274	A meta-analytic review of the hypoalgesic effects of exercise	Naugle et al.	2012	J Pain	Wrong study design
275	Efficacy of serious games for chronic pain management in older adults: A systematic review and metaâ€□analysis	Saragih et al.	2024	Journal of clinical nursing	Wrong study design
276	The effects of exercise on the quality of life of frail older adults: a preplanned meta-analysis of the FICSIT trials	Schechtman et al.	2001	Ann Behav Med	Wrong study design
277	The effectiveness of Pilates exercise in people with chronic low back pain: a systematic review	Wells et al.	2014	PLoS One	Wrong study design
278	Walking exercise for chronic musculoskeletal pain (PEDro synthesis)	Oliveira et al.	2016	British journal of sports medicine	Wrong study design
279	Effects of pilates on patients with chronic non-specific low back pain: a systematic review	Lin et al.	2016	Journal of physical therapy science	Wrong study design
280	Effectiveness of Tai Chi on older adults: A systematic review of systematic reviews with re-meta-analysis	Leung et al.	2022	Arch Gerontol Geriatr	Wrong study design
281	Exercise interventions on health-related quality of life for cancer survivors	Mishra et al.	2012	Cochrane Database Syst Rev	Wrong study design
282	Effect of Home Exercise Program in Patients with Knee Osteoarthritis: A Systematic Review and Meta-analysis	Anwer et al.	2016	Journal of Geriatric Physical Therapy	Wrong study design
283	Pilates for women with breast cancer: A systematic review and meta-analysis	Pinto-Carral et al.	2018	Complementary Therapies in Medicine	Wrong study design
284	Efficacy of aerobic exercise for treatment of chronic low back pain: a meta-analysis	Meng et al.	2015	American Journal of Physical Medicine & Rehabilitation	Wrong study design

285	The effects of Tai Chi on physical function and safety in patients with rheumatoid arthritis: A systematic review and meta-analysis	Wu et al.	2023	Front Physiol	Wrong study design
286	What Modifies the Effect of an Exercise Treatment for Chronic Low Back Pain? A Meta-epidemiologic Regression Analysis of Risk of Bias and Comparative Effectiveness	Niederer et al.	2022	J Orthop Sports Phys Ther	Wrong study design
287	Moderators of the effects of therapeutic exercise for people with knee and hip osteoarthritis: A systematic review of sub-group analyses from randomised controlled trials	Quicke et al.	2020	Osteoarthr Cartil Open	Wrong study design
288	The characteristics and effectiveness of pregnancy yoga interventions: a systematic review and meta-analysis	Corrigan et al.	2022	BMC Pregnancy Childbirth	Wrong study design
289	The effectiveness of exercise-based telemedicine on pain, physical activity and quality of life in the treatment of chronic pain: A systematic review	Adamse et al.	2018	J Telemed Telecare	Wrong study design
290	Effectiveness of exercise therapy on pain and quality of life of patients with primary dysmenorrhea: a systematic review with meta-analysis	Abaraogu et al.	2016	Turkish Journal of Physical Medicine & Rehabilitation / Turkiye Fiziksel Tip ve Rehabilitasyon Dergisi	Wrong study design
291	Physical activity interventions among adults with arthritis: meta-analysis of outcomes	Conn et al.	2008	Semin Arthritis Rheum	Wrong study design
292	Review: aerobic exercise improves cardiovascular fitness and tender points in fibromyalgia	Ciliska et al.	2003	Evidence Based Nursing	Wrong study design
293	Effect of Tai Chi on Quality of Life, Body Mass Index, and Waist-Hip Ratio in Patients With Type 2 Diabetes Mellitus: A Systematic Review and Meta-Analysis	Qin et al.	2020	Front Endocrinol (Lausanne)	Wrong study design
294	Are aquatic exercises efficacious in postmenopausal women with knee osteoarthritis? A meta-analysis of randomized controlled trials	Chen et al.	2019	The Journal of sports medicine and physical fitness	Wrong study design
295	Water-Based Exercise in Patients With Nonspecific Chronic Low-Back Pain: A Systematic Review With Meta-Analysis	Babiloni-Lopez et al.	2024	J Strength Cond Res	Wrong study design
296	Clinical relevance of Tai Chi on pain and physical function in adults with knee osteoarthritis: An ancillary meta-analysis of randomized controlled trials	Kelley et al.	2022	Sci Prog	Wrong study design

Supplementary content 3. Overview of all included systematic reviews and meta-analyses (n=158)

**Author, year**	**No. of RCTs**	**Total sample**	**Population or condition; gender; age mean (SD) or range (years)**	**Exercise mode, intensity and intervention duration.**	**Pain outcomes**
Aladro-Gonzalvo 2013, Costa Rica	9	242	Adults with non-specific low back pain<br>Females and males<br>Age: range 18 to 65y	Mode: Pilates.<br>Intensity: Low to moderate intensity.<br>Intervention duration: 4 to 12w.	Low back pain
Albuquerque 2022, Portugal	18	751	Adults with fibromyalgia<br>Females and males<br>Age: Ages 18+	Mode: Aerobic (including walking), resistance, mixed mode, pool-based exercises.<br>Intensity: Low to moderate.<br>Intervention duration: 8w to 8m.	Pain (general)
Al-Mhanna 2022, Malaysia	7	346	Patients with knee osteoarthritis<br>Females and males<br>Age: range 40 to 81y	Mode: Circuit training<br>Intensity: Low to vigorous<br>Intervention duration: 4w to 12m	Knee pain
Alzahrani 2019, Australia	3	422	Adults with non-specific low back pain<br>Females and males<br>Age: mean age range 46.7 to 51.6y	Mode: Walking<br>Intensity: Low to moderate<br>Intervention duration: 2m to 12m	Pain (short-term, intermediate-term, long-term)
Ariie 2023, Japan	52	3080	Adults with knee osteoarthritis<br>Females and males<br>Age: Ages >60y	Mode: Mixed mode exercise involving behaviour change techniques<br>Intensity: Low to vigorous<br>Intervention duration: 2w to 96m	Knee pain
Austin 2024, Australia	23	1954	Adults with cancer-related pain<br>Females and males<br>Age: mean (SD), 57.9 (8.5) y	Mode: Aerobic including walking, resistance and mixed mode.<br>Intensity: Low of moderate.<br>Intervention duration: 3w to 12m	Cancer-related pain
Baillet 2010, France	14	1040	Adults with Rheumatoid Arthritis<br>Females and males<br>Age: Mean age range, 44 to 68y	Mode: Aerobic exercise.<br>Intensity: Low to vigorous.<br>Intervention duration: 2w to 104w	Pain (general)
Baillet 2012, France	10	547	Adults with rheumatoid arthritis<br>Females and males<br>Age: Mean age range, 41 to 62y	Mode: Resistance exercise<br>Intensity: Low to vigorous<br>Intervention duration: 3w to 103w	Pain (general)
Barnet-Hepples 2024, Australia	26	4,403	Adults with chronic non-cancer pain<br>Females and males<br>Age: Mean age 56y	Mode: Physical activity (aerobic, resistance, mixed mode)<br>Intensity: Low to moderate<br>Intervention duration: 5w to 24m	Pain (general)
Barros Dos Santos 2021, Brazil	4	169	Adults with nonspecific chronic low back pain<br>Females and males<br>Age: range 20-65y	Mode: Aerobic, core stabilisation, Qigong<br>Intensity: Low to moderate<br>Intervention duration: 6w to 8w	Low-back pain
Bertozzi 2013, Italy	9	1063	Adults with chronic nonspecific neck pain<br>Females and males<br>Age: Mean 39y	Mode: Resistance, mixed mode, postural exercises<br>Intensity: Low to moderate<br>Intervention duration: 2w to 52w	Neck pain (short term, intermediate term)
Bhardwaj 2023, India	4	285	Adults with non-compressive neuropathic pain<br>Females and males<br>Age: Mean 30 to 62.9y	Mode: Yoga<br>Intensity: Low to moderate<br>Intervention duration: 2m to 3m	Neuropathic pain
Biswas 2024, UK	18	1402	Adults with osteoarthritis<br>Females and males<br>Age: Mean 57 to 79y	Mode: Yoga<br>Intensity: Low to moderate<br>Intervention duration: 1w to 6m	Pain (general)

Björk 2022, Sweden	55	4531	Adults with inflammatory arthritis<br>Females and males<br>Age: Mean 36.2y to 73.7y	Mode: Aerobic, resistance, mixed mode<br>Intensity: Low to vigorous<br>Intervention duration: 15d to 2y	Pain (general)
Busch 2007, Canada	16	724	Fibromyalgia<br>Females and males<br>Age: Mean 31y to 55y	Mode: Aerobic, resistance, mixed mode<br>Intensity: Low to vigorous<br>Intervention duration: 6w to 20w	Pain, tenderness
Busch 2013, Canada	5	241	Fibromyalgia<br>Females and males<br>Age: Mean 26y to 56y	Mode: Aerobic, resistance<br>Intensity: Low to vigorous<br>Intervention duration: 8w to 21w	Pain, tenderness
Busch 2002, Canada	34	2276	Fibromyalgia<br>Females and males<br>Age: Mean 27.5y to 60.2y	Mode: Aerobic, resistance, mixed mode<br>Intensity: Low to vigorous<br>Intervention duration: 6w to 2y	Pain, tenderness
Cheng 2019, Taiwan	6	657	Fibromyalgia<br>Females and males<br>Age: 48.3y to 54.8y	Mode: Tai Chi<br>Intensity: Low<br>Intervention duration: 10w to 24w	Pain (general)
Chen 2021A, China	5	251	Post-hip replacement<br>Females and males<br>Age: Mean 64.8y to 70.0y	Mode: Resistance.<br>Intensity: Low to moderate<br>Intervention duration: 6w to 12w	Hip pain
Chen 2021B, China	12	1430	Knee osteoarthritis and/or chronic knee pain<br>Females and males<br>Age: Mean 61.3y	Mode: Web-based, online, mobile app exercise interventions (mixed mode)<br>Intensity: Low to vigorous<br>Intervention duration: 4w to 6m	Knee pain
Chen 2016, Canada	24	1584	Cancer, osteoarthritis, heart failure or chronic obstructive pulmonary disease<br>Females and males<br>Age: Mean 65.9y	Mode: Tai Chi<br>Intensity: Low<br>Intervention duration: 6w to 24w	Pain (general)
Couto 2022, Portugal.	18	1184	Fibromyalgia<br>Females and males<br>Age: 18y and older	Mode: Aerobic, resistance, mixed mode<br>Intensity: Low to vigorous<br>Intervention duration: 3w to 24w	Pain (general)
Da Silva 2022, Brazil	9	443	Fibromyalgia<br>Females<br>Age: range 18 to 65y	Mode: Resistance<br>Intensity: Low to vigorous<br>Intervention duration: 8w to 12w	Pain (general)
De Almeida 2024, Brazil	11	434	Postmenopausal women<br>Females<br>Age: mean 54.1 to 73.2y	Mode: Pilates<br>Intensity: Low to moderate<br>Intervention duration: 6w to 52w	Bodily pain
Demaneuf 2019, Australia	10	389	Multiple Sclerosis<br>Females and males<br>Age: mean 31.6 to 54.6y	Mode: Aerobic, resistance, mixed mode, aquatic.<br>Intensity: Low to vigorous<br>Intervention duration: 5w to 26w	Pain (general)
Diez-Buil 2024, Spain	13	2488	Pregnant women with low back or pelvic pain<br>Females<br>Age: mean 29.2y	Mode: Pilates, Yoga, mixed mode<br>Intensity: Low to moderate<br>Intervention duration: 6w to 24w	Pain (general)
Duan 2022, China	19	1592	Knee or hip osteoarthritis<br>Females and males<br>Age: Mean 50.8 to 70.3y	Mode: Aquatic training<br>Intensity: Low to moderate<br>Intervention duration: 4w to 20w	Pain (general)
Du 2023, China	12	781	Fibromyalgia<br>Females and males<br>Age: mean 42.6 to 57.9y	Mode: Traditional Chinese exercises (Qigong, Tai Chi)<br>Intensity: Low to moderate<br>Intervention duration: 7w to 52w	Pain (general)
Fan 2024, Australia	13	2609	Hip or knee osteoarthritis<br>Females and males	Mode: Internet-delivered exercise<br>Intensity: Low to vigorous	Pain (general)

			Age: mean 56 to 65y	Intervention duration: 4w to 12m	
Fernandopulle 2017, New Zealand	27	3273	Hip and knee osteoarthritis<br>Females and males<br>Age: mean 54 to 73y	Mode: Aerobic, resistance, mixed mode, physical activity education<br>Intensity: Low to moderate<br>Intervention duration: 6m to 18m	Pain (general)
Ferraz 2023, Brazil	2	83	Pregnant women<br>Females<br>Age: mean 28 to 35y	Mode: Pilates<br>Intensity: Low to moderate<br>Intervention duration: 8w to 12 w	Low back pain
Fisseha 2016, Ethiopia	5	2087	Pregnant women<br>Females<br>Age: 18 years and older	Mode: Mixed mode, ergonomic exercise, individual and group based<br>Intensity: Low to moderate<br>Intervention duration: 4w to 12 w	Lumbopelvic pain
Fransen 2015, Australia	54	3913	Knee osteoarthritis<br>Females and males<br>Age: mean 53 to 75y	Mode: Aerobic, resistance, mixed mode<br>Intensity: Low to vigorous<br>Intervention duration: 4w to 12m	Pain (general)
#1380 – Fransen 2014, Australia	10	659	Hip osteoarthritis<br>Females and males<br>Age: mean 58y to 70y	Mode: Aerobic, resistance, mixed mode<br>Intensity: Low to vigorous<br>Intervention duration: 6w to 16w	Pain (general)
Gámez-Iruela 2024, Spain	11	951	Musculoskeletal diseases and chronic pain<br>Females and males<br>Age: Mean 71.5y	Mode: Yoga, Tai Chi, Pilates, Qigong, Mid-body exercises<br>Intensity: Low to moderate<br>Intervention duration: 4w to 12w	Chronic pain
Gilliam 2023, USA	8	558	Low back pain<br>Females and males<br>Age: 18 years and older	Mode: Pilates and yoga<br>Intensity: Low to moderate<br>Intervention duration: 6w to 12w	Pain (short and long term)
Guo 2022, China	7	668	Knee osteoarthritis<br>Females and males<br>Age: mean 58.5 to 71y	Mode: Mind body exercise (Qigong)<br>Intensity: Low to moderate<br>Intervention duration: 12w to 6m	Pain (general)
Guo 2023, China	16	1081	Cancer patients<br>Females and males<br>Age: range 44 to 72y	Mode: Mixed mode, aerobic<br>Intensity: Low to moderate<br>Intervention duration: 4w to 36w	Neuropathic pain
Guo 2022, China	15	1436	Knee osteoarthritis<br>Females and males<br>Age: mean 66y	Mode: Mixed mode, resistance<br>Intensity: Low to vigorous<br>Intervention duration: 4w to 72w	Pain (general)
Hall 2017, UK	15	1012	Musculoskeletal pain<br>Females and males<br>Age: 38y to 79y	Mode: Tai Chi<br>Intensity: Low to moderate<br>Intervention duration: 6w to 20w	Pain (short, medium, long term)<br>Low back pain (short, medium term)
Hall 2008, UK	19	717	Neurologic or Musculoskeletal Disease<br>Females and males<br>Age: Mean 56y	Mode: Aquatic exercise<br>Intensity: Low to moderate<br>Intervention duration: 4w to 20w	Pain (general)
Hall 2009, Australia	7	321	Musculoskeletal pain<br>Females and males<br>Age: mean 44 to 77y	Mode: Tai Chi<br>Intensity: Low to moderate<br>Intervention duration: 6w to 15w	Pain (general)
Han 2024, China	29	2071	Non-metastatic breast cancer<br>Females<br>Age: mean 42.1 to 78.8	Mode: Mixed mode<br>Intensity: Low to vigorous<br>Intervention duration: 4w to 8m	Pain (general)
Hansen 2020, Denmark	3	189	Hip osteoarthritis<br>Females and males<br>Age: 50 years and older	Mode: Resistance<br>Intensity: Low to vigorous<br>Intervention duration: 6w to 4m	Pain (general)
Hart 2019, USA	16	1254	Older adults<br>Females and males<br>Age: 59.9 to 82.1	Mode: Resistance<br>Intensity: Low to vigorous<br>Intervention duration: 8w to 104w	Bodily pain

Häuser 2010, Germany	35	2494	Fibromyalgia<br>Females and males<br>Age: mean 45	Mode: Aerobic, Mixed mode<br>Intensity: Low to vigorous<br>Intervention duration: 6w to 104w	Pain (general)
Hayden 2020, Canada	27	3514	Low back pain<br>Females and males<br>Age: mean 37 to 60.1	Mode: Aerobic, resistance, mixed mode, yoga.<br>Intensity: Low to vigorous<br>Intervention duration: ≥6 weeks	Pain (general)
Heidari 2023, Iran	14	484	Low back pain<br>Females and males<br>Age: mean 47.3 y	Mode: Aquatic exercise<br>Intensity: Low to moderate<br>Intervention duration: 6w to 4m	Pain (general)
Hernandez-Lucas 2022, Spain	4	1235	Low back pain<br>Females and males<br>Age: mean 46y	Mode: Resistance, yoga<br>Intensity: Low to moderate<br>Intervention duration: 3w to 9w	Pain (general)
Hernández-Molina 2008, USA	9	1,234	Hip Osteoarthritis<br>Females and males<br>Age: 18 years and older	Mode: Mixed mode, tai chi, aquatic<br>Intensity: Low to moderate<br>Intervention duration: 6w to 26w	Pain (general)
Holden 2023, UK	31	4241	Knee and hip osteoarthritis<br>Females and males<br>Age: Mean 57y to 79y	Mode: Aerobic, resistance, mixed mode, Tai Chi, aquatic, web-based PA interventions<br>Intensity: Low to vigorous<br>Intervention duration: 4w to 104w	Pain (short term, medium term)
Holtzman 2013, Canada	8	743	Low back pain<br>Females and males<br>Age: mean 48.3 to 49	Mode: Yoga<br>Intensity: Low<br>Intervention duration: 1w to 16w	Pain (general)
Hsueh 2021, Taiwan	26	2069	Breast cancer<br>Females<br>Age: 18-75	Mode: Yoga<br>Intensity: Low<br>Intervention duration: 1w to 6m	Pain (general)
Huang 2023, China	15	1124	Cancer patients with peripheral neuropathy<br>Females and males<br>Age: 44 to 68.5	Mode: Aerobic, resistance, mixed mode, Yoga<br>Intensity: Low to moderate<br>Intervention duration: 2w to 36w	Pain (general)
Hu 2021, China	16	986	Knee osteoarthritis<br>Females and males<br>Age: 59.9 to 78.9y	Mode: Tai Chi<br>Intensity: Low<br>Intervention duration: 5w to 1y	Pain (general)
Hu 2020, China	10	534	Ankylosing spondylitis<br>Females and males<br>Age: 18 years and older	Mode: Aerobic, resistance, mixed mode, Pilates, Tai Chi<br>Intensity: Low to moderate<br>Intervention duration: 8w to 24 weeks	Pain (general)
Ji-Ah 2022, Korea	20	1609	Osteoarthritis<br>Females and males<br>Age: 48 to 75y	Mode: Aquatic exercise<br>Intensity: Low to moderate<br>Intervention duration: 6w to 24w	Pain (general)
Jones 2022, Australia	11	743	Adults Aged 65 years and older<br>Females and males<br>Age: 66 to 74.8	Mode: Bandujin exercise:<br>Intensity: Low<br>Intervention duration: 3d to 12m	Pain (general)
Juhl 2014, Denmark	48	4092	Knee osteoarthritis<br>Females and males<br>Age: mean 64.3	Mode: Aerobic, resistance, mixed mode, Yoga and traditional Chinese exercise<br>Intensity: Low to moderate<br>Intervention duration: 3w to 18m	Pain (general)
Jurado-Castro 2022, Spain	6	410	Patients with overweight or obesity suffering from knee osteoarthritis<br>Females and males<br>Age: mean 64y	Mode: Aerobic, resistance, mixed mode,<br>Intensity: Low to vigorous<br>Intervention duration: 1m to 18m	Pain (general)
Kang 2011, Korea	9	521	Osteoarthritis	Mode: Tai Chi	Pain (general)

			Females and males<br>Age: mean 59.9 to 72.8	Intensity: Low<br>Intervention duration: 8w to 6m	
Kelley 2011, USA	33	3180	Osteoarthritis, rheumatoid arthritis, Fibromyalgia<br>Females and males<br>Age: mean 57y	Mode: Aerobic, resistance, mixed mode, aquatic, Tai Chi<br>Intensity: Low to vigorous<br>Intervention duration: 6w to 78w	Pain (general)
Kim 2019, Korea	4	230	Primary dysmenorrhea<br>Females<br>Age: mean 20.0y to 33.6	Mode: Yoga<br>Intensity: Low to moderate<br>Intervention duration: 2w to 12w	Pain (general)
Kim 2020, Korea	6	532	Low back pain<br>Females and males<br>Age: range 18-65y	Mode: Yoga<br>Intensity: Low to moderate<br>Intervention duration: 12w	Pain (general)
Kong 2016, China	18	1260	Chronic pain conditions<br>Females and males<br>Age: Mean 61.4y	Mode: Tai Chi<br>Intensity: Moderate<br>Intervention duration: 6w to 28w	Pain (osteoarthritis, low back pain, osteoporosis, fibromyalgia)
Kong 2022, China	21	1770	Neck pain<br>Females and males<br>Age: mean 49.6y	Mode: Traditional Chinese exercise<br>Intensity: Low to moderate<br>Intervention duration: 4w to 24w	Paín (general)
Langhorst 2013, Germany	7	362	Fibromyalgia<br>Females and males<br>Age: Mean 50y	Mode: Yoga, Tai Chi, Qigong<br>Intensity: Low<br>Intervention duration: 4 to 12w	Pain (general)
LaTouche 2020, Spain	10	508	Patients with migraine<br>Females and males<br>Age: Mean 31 to 47y	Mode: Aerobic<br>Intensity: Low to vigorous<br>Intervention duration: 6 to 12w	Pain (general)
Lauche 2019, Australia	9	640	Osteoarthritis<br>Females and males<br>Age: range 50 to 80y	Mode: Yoga<br>Intensity: Low to moderate<br>Intervention duration: 8w to 12w	Pain (general)
Lauche 2013, Germany	5	252	Knee osteoarthritis<br>Females and males<br>Age: >50y	Mode: Tai Chi<br>Intensity: Low<br>Intervention duration: 8w to 20w	Pain (short term. Long term)
Lawrence 2017, UK	2	72	Stroke<br>Females and males<br>Age: Mean 56.1 to 63.9	Mode: Yoga<br>Intensity: Low<br>Intervention duration: 8w to 10w	Pain (general)
Lezheng 2023, China	12	938	Cancer<br>Females and males<br>Age: mean 53.9y	Mode: High-intensity interval training<br>Intensity: Vigorous<br>Intervention duration: 5w to 24w	Cancer-related pain
Liang 2019, China	10	871	Neck pain<br>Females and males<br>Age: Mean 23.1 to 50.8y	Mode: Resistance exercise, Qigong (Baduanjin)<br>Intensity: Low to moderate<br>Intervention duration: 10d to 8w	Pain (general)
Li 2023, China	39	2325	Osteoporosis<br>Female only<br>Age: mean 51.8 to 73.9	Mode: Mind body exercise (Yoga, Pilates, Tai Chi), dance<br>Intensity: Low to moderate<br>Intervention duration: 2.5 to >12m	Pain (general)
Lim 2011, Singapore	7	194	Low Back Pain<br>Females and males<br>Age: 18y and older	Mode: Pilates<br>Intensity: Low to moderate<br>Intervention duration: 6w to 8w	Pain (general)
#2412 – Li 2020, China	14	815	Knee osteoarthritis<br>Females and males<br>Age: mean 56.1 to 70.8	Mode: Traditional Chinese Exercise (Tai Chi, Qigong)<br>Intensity: Low<br>Intervention duration: 4w to 24w	Pain (general)

Liu 2009, USA	121	6700	Older adults<br>Females and males<br>Age:60 y and older	Mode: Resistance<br>Intensity: Moderate to vigorous<br>Intervention duration: 8w to >12w	Pain (general)
Li 2009, Taiwan	4	256	Osteoporotic and osteopenic postmenopausal<br>Females<br>Age: 50 years and older	Mode: Aerobic, resistance, mixed mode<br>Intensity: Low to vigorous<br>Intervention duration: 10w to 25w	Pain (general)
#2424 – Li 2023, China	12	1289	Osteoporotic vertebral fracture<br>Females and males<br>Age: mean 58.9 to 74.7	Mode: Resistance and balance<br>Intensity: Low to moderate<br>Intervention duration: 4w to 1y	Pain (general)
#2434 – Li 2016, China	17	1705	Knee osteoarthritis<br>Females and males<br>Age: mean 63.5y	Mode: Resistance exercise<br>Intensity: Low to vigorous<br>Intervention duration: 8w to 24m	Pain (general)
Long 2022, China	6	445	Patients with migraine<br>Females and males<br>Age: mean 30.5 to 44y	Mode: Yoga<br>Intensity: Low to moderate<br>Intervention duration: NR	Migraine
Luan 2021, China	11	724	Knee osteoarthritis<br>Females and males<br>Age: range 40 to 8 y	Mode: Aerobic (stationary cycling)<br>Intensity: Low to vigorous<br>Intervention duration: 4w to 12w	Pain (general)
Lu 2024, China	8	756	Knee osteoarthritis<br>Females and males<br>Age: mean 51 to 76.4y	Mode: Yoga<br>Intensity: Low to moderate<br>Intervention duration:	Pain (general)
Luo 2020, China	15	885	Breast Cancer Patients<br>Females<br>Age: range 45-60y	Mode: Tai Chi<br>Intensity: Low<br>Intervention duration: 12w to 6m	Pain (3, 6, 12 weeks)
Mahmoudi 2022, Iran	80	1345	Healthy Older Adults<br>Females and males<br>Age: 65.2 to 85.4y	Mode: Aerobic, resistance, mixed mode<br>Intensity: Low to vigorous<br>Intervention duration: 8w to 36w	Bodily pain
Ma 2018, China	9	777	Patients undergoing total knee arthroplasty<br>Females and males<br>Age: mean 62.6 to 72.8y	Mode: Aerobic, resistance, mixed mode, aquatic training<br>Intensity: Low to moderate<br>Intervention duration: 4w to 12 w	Bodily pain
Mao 2024, China	16	3015	Hip and knee osteoarthritis<br>Females and males<br>Age: mean 55 to 74y	Mode: Home-based exercise<br>Intensity: Low to moderate<br>Intervention duration: 8w to 12m	Pain (general)
Meender Schäfer 2018, Germany	7	742	Knee osteoarthritis<br>Females and males<br>Age: 50y and older	Mode: Aerobic, resistance, mHealth and telehealth supported physical activity.<br>Intensity: Low to moderate<br>Intervention duration: 6w to 18m	Pain (short term, long term)
Miller 2022, Australia	79	4843	Chronic primary musculoskeletal pain<br>Females and males<br>Age: mean 20 to 76 years	Mode: Aerobic, resistance, mixed mode, exergames, aquatic exercise, Tai Chi, Pilates, Yoga<br>Intensity: Low to vigorous<br>Intervention duration: 2w to 104w	Chronic pain
Miyamoto 2013, Brazil	8	190	Low back pain<br>Females and males<br>Age: mean 21.6 to 44.1	Mode: Pilates<br>Intensity: Low to moderate<br>Intervention duration: 4w to 8w	Pain (general)
Moseng 2017, Norway	12	1202	Hip osteoarthritis<br>Females and males<br>Age: mean 66	Mode: Aerobic, resistance, mixed mode<br>Intensity: Low to moderate<br>Intervention duration: 5w to 12w	Pain (general)

Murillo-García 2018, Spain	7	335	Fibromyalgia<br>Females and males<br>Age: mean 49.1 to 57.0	Mode: Dance<br>Intensity: Low to moderate<br>Intervention duration: 12w to 24w	Pain (general)
Nakano 2018, Japan	10	893	Cancer<br>Females and males<br>Age: mean 41.4 to 70.1	Mode: Aerobic, resistance, mixed mode.<br>Intensity: Low to vigorous<br>Intervention duration: 3w to 16w	Pain (general)
Nunan 2022, UK	11	622	Irritable bowel syndrome<br>Females and males<br>Age: Mean range 19.0 to 66.5	Mode: Aerobic, resistance, mixed mode, Qigong, Yoga,<br>Intensity: Low to moderate<br>Intervention duration: 6w to 24w	Abdominal pain
Oliveira 2018, Australia	4	180	Chronic musculoskeletal pain<br>Females and males<br>Age: Mean 33.5 to 58.0 years	Mode: Aerobic, dance<br>Intensity: Low to moderate<br>Intervention duration: 8w to 24w	Pain (short term, immediate term)
Pedersen 2023, Denmark	35	2412	Knee osteoarthritis<br>Females and males<br>Age: 62.1y	Mode: Aerobic, resistance, mixed mode<br>Intensity: Low to vigorous<br>Intervention duration: 4w to 36w	Pain (general)
Pereira 2012, Brazil	5	139	Low back pain<br>Females and males<br>Age: 18 years and older	Mode: Pilates<br>Intensity: Low to moderate<br>Intervention duration: 4w to 7.3w	Pain (general)
Plinsinga 2023, Australia	71	5877	Cancer<br>Females and males<br>Age: mean 57	Mode: Aerobic, resistance, mixed mode, yoga<br>Intensity: Low to vigorous<br>Intervention duration: 2w to 12m	Pain (general)
Polaski 2019, USA	75	2721	Chronic pain<br>Females and males<br>Age: 18 years and older	Mode: Aerobic, resistance, mixed mode, Qigong, Tai Chi, aquatic exercise, Pilates<br>Intensity: Low to vigorous<br>Intervention duration: 4w to 104w	Pain (general)
Ponzano 2021, Canada	53	4618	Low bone mineral density, osteopenia or osteoporosis<br>Females and males<br>Age: 50 years and older	Mode: Resistance<br>Intensity: Low to vigorous<br>Intervention duration: 4w to 30m	Pain (general)
Qian 2020, China	13	1306	Breast cancer<br>Females<br>Age: mean 39 to 59 y	Mode: Aerobic, resistance, mixed mode, Yoga, Qigong, Nia exercise<br>Intensity: Low to vigorous<br>Intervention duration: 4w to 12w	Pain (general)
Qiao 2024, China	17	1122	Knee osteoarthritis<br>Females and males<br>Age: mean 60.3 to 78.9	Mode: Mind body exercise<br>Intensity: Low to moderate<br>Intervention duration: 8w to 24w	Pain (general)
Qin 2021, China	18	1418	Type 2 diabetes mellitus<br>Females and males<br>Age: 47.8 to 68	Mode: Tai Chi<br>Intensity: Low<br>Intervention duration: 12w to 24w	Pain (general)
Qin 2019, China	10	959	Low back pain<br>Females and males<br>Age: mean 32.6y	Mode: Tai Chi<br>Intensity: Low<br>Intervention duration: 2w to 28w	Pain (general)
Quentin 2021, France	53	9588	Low back pain<br>Females and males<br>Age: mean 49.3	Mode: Aerobic, resistance, mixed mode<br>Intensity: Low to vigorous<br>Intervention duration: 2w to 2y	Pain (general)
Ramel 2009, USA	10	767	Fibromyalgia<br>Females and males<br>Age: 47	Mode: Aerobic, resistance, mixed mode, aquatic exercise<br>Intensity: Low to vigorous<br>Intervention duration: 6w to 24w	Pain (general)
Roberts 2020, Australia	7	400	Breast cancer<br>Females	Mode: Aerobic, resistance, mixed mode<br>Intensity: Low to moderate	Pain (general)

			Age: mean range: 61 to 63.8 y	Intervention duration: 6w to 12m	
Roddy 2005, UK	13	2304	Knee osteoarthritis<br>Females and males<br>Age: 61.9 to 86.6	Mode: Aerobic, resistance<br>Intensity: Low to moderate<br>Intervention duration: 8w to 2y	Pain (general)
Rodríguez-Almagro 2023, Spain	15	894	Fibromyalgia<br>Females<br>Age: mean 38 to 59y	Mode: Resistance<br>Intensity: Low to vigorous<br>Intervention duration: 8w to 21w	Pain (general)
Sánchez-Polán 2023, Spain	16	2613	Pregnant women<br>Females<br>Age: 18 years and older	Mode: Aerobic, resistance, mixed mode, Pilates, Yoga,<br>Intensity: Low to moderate<br>Intervention duration: 4w to 22w	Pain (general)
Saragiotto 2020, Brasil	12	1456	Musculoskeletal Disorders<br>Females and males<br>Age: mean 45.3 to 72.9	Mode: Aerobic (walking)<br>Intensity: Low to moderate<br>Intervention duration: 4w to 12m	Pain (short term, intermediate term, long term)
Sasaki 2022, Japan	20	2350	Hip and knee osteoarthritis<br>Females and males<br>Age: mean 55.5y to 73.7	Mode: Aerobic, resistance, mixed mode, physical activity interventions<br>Intensity: Low to moderate<br>Intervention duration: 6w to 24m	Pain (general)
Shen 2020, China	13	1306	Breast cancer<br>Females<br>Age: 39.2 to 59	Mode: Aerobic, resistance, mixed mode, Qigong, Yoga, Nia exercise<br>Intensity: Low to moderate<br>Intervention duration: NR	Pain (general)
Shiri 2018, Finland	11	2347	Pregnant women with low back pain<br>Females<br>Age: NR	Mode: Aerobic, resistance, mixed mode, gymnastics,<br>Intensity: Low to moderate<br>Intervention duration: 8w to 8m	Pain (low back, pelvic girdle, lumbopelvic)
Sobue 2022, Japan	9	1343	Rheumatoid arthritis<br>Females and males<br>Age: mean 48.6 to 64.	Mode: Aerobic, resistance, mixed mode, aquatic exercise,<br>Intensity: Low to moderate<br>Intervention duration: 2w to 16w	Pain (general)
Tanaka 2014, Japan	17	1635	Knee osteoarthritis<br>Females and males<br>Age: mean 27 to 29	Mode: Aerobic, Resistance, Tai Chi. Qigong<br>Intensity: Low to moderate<br>Intervention duration: 6w to 72w	Pain (general)
Tanaka 2013, Japan	33	3,192	Knee osteoarthritis<br>Females and males<br>Age: range: mean 56.8 to 73.9	Mode: Aerobic, Resistance, Mixed mode Tai Chi. Qigong<br>Intensity: Low to moderate<br>Intervention duration: 6w to 72w	Pain (VAS and WOMAC)
Tan 2024, China	33	2621	Knee osteoarthritis<br>Females and males<br>Age: mean 53.4 to 77.4	Mode: Traditional Chinese Exercise (Tai Chi, Qigong)<br>Intensity: Low<br>Intervention duration: 3w to 6m	Pain (general)
Tanriverdi 2023, Turkey	14	1034	Cancer (palliative care)<br>Females and males<br>Age: mean 52 to 70 years	Mode: Aerobic, resistance, mixed mode<br>Intensity: Low to moderate<br>Intervention duration: 2w to 12w	Pain (general)
Teirlinck 2023, Netherlands	18	1345	Hip osteoarthritis<br>Females and males<br>Age: 18 years and older	Mode: Aerobic, resistance, mixed mode, aquatic exercise<br>Intensity: Low to moderate<br>Intervention duration: 5w to 16w	Pain (general)
Umehara 2018, Japan	27	2432	Knee osteoarthritis<br>Females and males<br>Age: mean 60.6 to 74.6 years	Mode: Aerobic, resistance, mixed mode, aquatic exercise<br>Intensity: Low to moderate<br>Intervention duration: 4w to 8w	Pain (preoperative, post-operative)
Varangot-Reille 2022, Spain	19	2776	Migraine or Tension-Type Headache<br>Females and males<br>Age: mean 31 and 47 years	Mode: Aerobic, resistance, mixed mode<br>Intensity: Low to moderate<br>Intervention duration: 3w to 6m	Migraine, tension-type headache
Verville 2023, Canada	13	1362	Low back pain<br>Females and males<br>Age: 37 to 74 years	Mode: Aerobic, resistance, mixed mode, Pilates, Yoga<br>Intensity: Low to moderate<br>Intervention duration: 2w to 12m	Pain (general)

Vilarino 2023, Brazil	13	839	Fibromyalgia<br>Females and males<br>Age: mean 50.7y	Mode: Resistance<br>Intensity: Low to vigorous<br>Intervention duration: 8w to 21w	Pain (general)
Waller 2014, Finland	11	1902	Hip and knee osteoarthritis<br>Females and males<br>Age: mean 61.5 to 75.8	Mode: Aquatic exercise<br>Intensity: Low to moderate<br>Intervention duration: 6w to 52w	Pain (general)
Wang 2024, Taiwan	11	530	Fibromyalgia<br>Females and males<br>Age: Mean 37 to 60.2	Mode: Resistance<br>Intensity: Low to vigorous<br>Intervention duration: 8w to 21w	Pain (general)
Wang Susu 2021, China	33	1481	Patients undergoing maintenance haemodialysis<br>Females and males<br>Age: mean 42 to 67	Mode: Intradialytic exercise (aerobic, resistance, mixed mode)<br>Intensity: Low to moderate<br>Intervention duration: 8w to 6m	Pain (general)
Wang 2023, China	32	2200	Chronic musculoskeletal disorders<br>Females and males<br>Age: 44.6 to 68	Mode: Aquatic exercise<br>Intensity: Low to moderate<br>Intervention duration: 3w to 20w	Pain (Osteoarthritis, Fibromyalgia, Low back pain)
Wang 2018, China	13	1557	Knee arthritis<br>Females and males<br>Age: 18 years and older	Mode: Yoga<br>Intensity: Low<br>Intervention duration: 1w to 10w	Pain (general)
Wang 2017, China	5	344	Perimenopause<br>Females<br>Age: range 45 to 70y	Mode: Tai Chi<br>Intensity: Low<br>Intervention duration: 20w to 26w	Bodily pain
Wei 2024, China	9	456	Knee osteoarthritis<br>Females and males<br>Age: mean 22.6 to 77.6y	Mode: Virtual reality exercise<br>Intensity: Low<br>Intervention duration: 3w to 12w	Pain (general)
Wei 2015, China	4	444	Osteoporosis<br>Females and males<br>Age: 55.0 to 69.2	Mode: Qigong (Wuqinxi)<br>Intensity: Low<br>Intervention duration: 6m to 12m	Pain (general)
Wen 2022, China	17	1332	Middle and older adults with chronic pain<br>Females and males<br>Age: mean 56.1 to 75.8	Mode: Mind body exercise (Tai Chi. Qigong, Yoga)<br>Intensity: Low<br>Intervention duration: 8w to 24w	Pain (general)
Wewege 2018, Australia	6	333	Low back pain<br>Females and males<br>Age: Mean 43.6y	Mode: Aerobic, resistance<br>Intensity: Low to vigorous<br>Intervention duration: 6w to 16w	Pain (general)
Wu 2022, China	9	466	Fibromyalgia<br>Females<br>Age: mean 29.5 to 55.6	Mode: Exergames<br>Intensity: Low to moderate<br>Intervention duration: 6w to 24w	Pain (general)
Wu 2022, China	5	356	Migraine<br>Females and males<br>Age: mean 30.5 to 36.8	Mode: Yoga<br>Intensity: Low<br>Intervention duration: NR	Headache frequency, headache intensity, pain
Yamato 2016, Australia	10	510	Low back pain<br>Females and males<br>Age: Range 18 to 70y	Mode: Pilates<br>Intensity: Low to moderate<br>Intervention duration: 4w to 8w	Pain (short term, intermediate term)
Yang 2022, Australia	37	3525	Cardiovascular disease<br>Females and males<br>Age: 40 years and older	Mode: Tai Chi<br>Intensity: Low<br>Intervention duration: 8w to 240w	Pain (general)
Yang 2023, China	9	861	Knee osteoarthritis<br>Females and males<br>Age: Mean 53.7 to 72.2 years	Mode: Telehealth (aerobic, resistance, mixed mode)<br>Intensity: Low to moderate<br>Intervention duration: 6w to 6m	Pain (general)

Yang 2022, China	7	242	Haematological cancers<br>Females and males<br>Age: 18 years and older	Mode: Aerobic, resistance<br>Intensity: Low to moderate<br>Intervention duration: NR	Pain (general)
Yan 2013, China	7	348	Osteoarthritis<br>Females and males<br>Age: mean 59.9 to 7.80	Mode: Tai Chi<br>Intensity: Low<br>Intervention duration: 8w to 24w	Pain (general)
Yan 2021, China	20	1824	Osteoporosis<br>Females<br>Age: mean 50.2 to 70.3	Mode: Aerobic, resistance, mixed mode, Tai Chi, Qigong, Mountaineering,<br>Intensity: Low to vigorous<br>Intervention duration: 12w to 14m	Pain (general)
Ye 2022, China	13	967	Rheumatoid arthritis<br>Females and males<br>Age: mean 45.0 to 70.1	Mode: Aerobic<br>Intensity: Low to vigorous<br>Intervention duration: 2w to 48w	Pain (general)
Ye 2020, China	10	840	Rheumatoid arthritis<br>Females and males<br>Age: mean 27.1 to 66.7	Mode: Yoga<br>Intensity: Low<br>Intervention duration: 40d to 12w	Pain (general)
Yilmaz 2023, Turkey	4	201	Pregnancy<br>Females<br>Age: mean 25.2 to 29y	Mode: Pilates<br>Intensity: Low to moderate<br>Intervention duration: 6w to 8w	Pain (pregnancy, labour)
Zeng 2020, China	7	424	Knee osteoarthritis<br>Females and males<br>Age: mean 55.8 to 71.6y	Mode: Qigong<br>Intensity: Low<br>Intervention duration: 8y to 1y	Pain (general)
Zhang 2022, China	7	296	Lumbar spondylosis and back pain<br>Females and males<br>Age: mean 32.6 to 58.9	Mode: Tai Chi<br>Intensity: Low<br>Intervention duration: NR	Pain (general)
Zhang 2023, China	5	581	Pregnancy<br>Females<br>Age: 18 years and older	Mode: Yoga<br>Intensity: Low<br>Intervention duration: 6w to 12w	Pain (general)
Zhang 2024, China	20	1670	Axial spondyloarthritis<br>Females and males<br>Age: mean 44.4	Mode: aerobic, resistance, mixed mode<br>Intensity: Low to vigorous<br>Intervention duration: 6w to 24w	Pain (general)
Zhang 2023, China	18	989	Low back pain<br>Females and males<br>Age: mean 57 to 73	Mode: Aerobic, resistance, aquatic exercise, Yoga, Qigong, Pilates, Virtual reality training,<br>Intensity: Low to vigorous<br>Intervention duration: 4w to 21w	Pain (general)
Zhang 2023, China	17	1174	Knee osteoarthritis<br>Females and males<br>Age: 53.4 to 71	Mode: Traditional Chinese exercise<br>Intensity: Low<br>Intervention duration: 8w to 24w	Pain (general)
Zhang 2023, China	22	1931	Lumbar disc herniation<br>Females and males<br>Age: mean 40.6 to 59.9	Mode: Traditional Chinese exercise<br>Intensity: Low<br>Intervention duration: NR	Pain (general)
Zhang 2017, China	8	375	Knee osteoarthritis<br>Females and males<br>Age: mean 62.5 to 70.8	Mode: Traditional Chinese exercise<br>Intensity: Low<br>Intervention duration: 8w to 48w	Pain (general)
Zhang 2019, China	11	886	Low back pain<br>Females and males<br>Age: mean 35.2 to 74	Mode: Traditional Chinese exercise<br>Intensity: Low<br>Intervention duration: 2w to 24w	Pain (general)
Zhang 2019, China	15	857	Osteoporosis<br>Females and males<br>Age: mean 55.5 to 71.3	Mode: Tai Chi<br>Intensity: Low<br>Intervention duration: 16w to 12m	Pain (general)

Zhou 2019, China	23	1235	Type 2 diabetes<br>Females and males<br>Age: mean 35 to 70.4	Mode: Tai Chi<br>Intensity: Low<br>Intervention duration: 4w to 24w	Pain (general)
Zhu 2020, China	18	1863	Low back pain<br>Females and males<br>Age: mean 33.6 to 73	Mode: Yoga<br>Intensity: Low<br>Intervention duration: 1w to 24w	Pain (general)
Zou 2019, China	17	1932	Low back pain<br>Females and males<br>Age: mean 34 to 74	Mode: Mind-body exercise (Yoga, Tai Chi, Qigong)<br>Intensity: Low<br>Intervention duration: 1w to 24w	Pain (general)

Supplementary content 4. AMSTAR-2 risk of bias and study quality ratings.

	AMSTAR 2 Item																Overall
**Author, year**	**1**	**2**	**3**	**4**	**5**	**6**	**7**	**8**	**9**	**10**	**11**	**12**	**13**	**14**	**15**	**16**	
Aladro-Gonzalvo 2013	Yes	No	Yes	PY	No	Yes	No	Yes	Yes	No	Yes	Yes	Yes	Yes	NA	Yes	Critically low
Albuquerque 2022	Yes	PY	Yes	PY	Yes	No	No	Yes	Yes	No	Yes	Yes	Yes	Yes	No	Yes	Critically low
Al-Mhanna 2022	Yes	PY	Yes	PY	No	No	No	Yes	Yes	No	Yes	Yes	Yes	Yes	NA	Yes	Low
Alzahrani 2019	Yes	PY	Yes	PY	Yes	Yes	No	Yes	Yes	No	Yes	Yes	Yes	Yes	NA	Yes	Critically low
Ariie 2023	Yes	PY	Yes	Yes	Yes	Yes	Yes	Yes	Yes	No	Yes	Yes	Yes	Yes	Yes	Yes	High
Austin 2024	Yes	PY	Yes	Yes	Yes	Yes	No	Yes	Yes	No	Yes	Yes	Yes	Yes	Yes	Yes	Low
Baillet 2010	Yes	No	Yes	PY	No	Yes	No	Yes	Yes	No	Yes	Yes	Yes	Yes	Yes	Yes	Critically low
Baillet 2012	Yes	No	Yes	PY	Yes	Yes	No	Yes	Yes	No	Yes	Yes	Yes	Yes	Yes	Yes	Critically low
Barnet-Hepples 2024	Yes	PY	Yes	Yes	Yes	Yes	No	Yes	Yes	No	Yes	Yes	Yes	Yes	Yes	Yes	Low
Barros Dos Santos 2021	Yes	PY	Yes	PY	Yes	Yes	No	Yes	Yes	No	Yes	Yes	Yes	Yes	NA	Yes	Low
Bertozzi 2013	Yes	No	Yes	PY	Yes	Yes	No	Yes	Yes	No	Yes	Yes	Yes	Yes	NA	Yes	Critically low
Bhardwaj 2023	Yes	PY	Yes	Yes	No	No	No	Yes	Yes	No	Yes	Yes	Yes	Yes	NA	Yes	Low
Biswas 2024	Yes	PY	Yes	Yes	Yes	Yes	Yes	Yes	Yes	No	Yes	Yes	Yes	Yes	No	Yes	Low
Björk 2022	Yes	PY	Yes	PY	Yes	Yes	Yes	Yes	Yes	No	Yes	Yes	Yes	Yes	Yes	Yes	High
Busch 2007	Yes	Yes	Yes	PY	Yes	Yes	Yes	Yes	Yes	Yes	Yes	Yes	Yes	Yes	NA	Yes	High
Busch 2013	Yes	No	Yes	PY	Yes	Yes	No	Yes	Yes	No	Yes	Yes	Yes	Yes	No	Yes	Critically low
Busch 2002	Yes	Yes	Yes	PY	Yes	Yes	Yes	Yes	Yes	Yes	Yes	Yes	Yes	Yes	No	Yes	Low
Cheng 2019	Yes	PY	Yes	PY	No	Yes	No	Yes	Yes	No	Yes	Yes	Yes	Yes	NA	Yes	Low
Chen 2021A	Yes	PY	Yes	PY	No	Yes	No	Yes	Yes	No	Yes	Yes	Yes	Yes	NA	Yes	Low
Chen 2021B	Yes	PY	Yes	PY	Yes	Yes	No	Yes	Yes	No	Yes	Yes	Yes	Yes	Yes	Yes	Low
Chen 2016	Yes	PY	Yes	PY	Yes	Yes	No	Yes	Yes	No	Yes	Yes	Yes	Yes	Yes	Yes	Low
Couto 2022	Yes	PY	Yes	PY	Yes	No	No	Yes	Yes	No	Yes	Yes	Yes	Yes	No	Yes	Critically low
Da Silva 2022	Yes	PY	Yes	PY	Yes	Yes	No	Yes	Yes	No	Yes	Yes	Yes	Yes	Yes	Yes	Low

De Almeida 2024	Yes	PY	Yes	PY	Yes	Yes	No	Yes	Yes	No	Yes	Yes	Yes	Yes	No	Yes	Critically low
Demaneuf 2019	Yes	PY	Yes	PY	Yes	Yes	No	Yes	Yes	No	Yes	Yes	Yes	Yes	Yes	Yes	Low
Diez-Buil 2024	Yes	PY	Yes	Yes	Yes	Yes	No	Yes	Yes	No	Yes	Yes	Yes	Yes	No	Yes	Critically low
Duan 2022	Yes	PY	Yes	PY	Yes	Yes	No	Yes	Yes	No	Yes	Yes	Yes	Yes	Yes	Yes	Low
Du 2023	Yes	PY	Yes	PY	Yes	Yes	No	Yes	Yes	No	Yes	Yes	Yes	Yes	Yes	Yes	Low
Fan 2024	Yes	PY	Yes	PY	Yes	Yes	No	Yes	Yes	No	Yes	Yes	Yes	Yes	No	Yes	Critically low
Fernandopulle 2017	Yes	PY	Yes	PY	Yes	No	No	Yes	Yes	No	Yes	Yes	Yes	Yes	No	Yes	Critically low
Ferraz 2023	Yes	PY	Yes	PY	Yes	Yes	No	Yes	Yes	No	Yes	Yes	Yes	Yes	NA	Yes	Low
Fisseha 2016	Yes	PY	Yes	PY	No	No	No	Yes	Yes	No	Yes	Yes	Yes	Yes	NA	Yes	Low
Fransen 2015	Yes	PY	Yes	PY	Yes	Yes	Yes	Yes	Yes	No	Yes	Yes	Yes	Yes	No	Yes	Low
Fransen 2014	Yes	PY	Yes	PY	Yes	Yes	Yes	Yes	Yes	No	Yes	Yes	Yes	Yes	NA	Yes	High
Gámez-Iruela 2024	Yes	Yes	Yes	PY	Yes	Yes	Yes	Yes	Yes	Yes	Yes	Yes	Yes	Yes	Yes	Yes	High
Gilliam 2023	Yes	PY	Yes	PY	Yes	Yes	No	Yes	Yes	No	Yes	Yes	Yes	Yes	NA	Yes	Low
Guo 2022	Yes	PY	Yes	PY	Yes	Yes	No	Yes	Yes	No	Yes	Yes	Yes	Yes	NA	Yes	Low
Guo 2023	Yes	PY	Yes	Yes	Yes	Yes	No	Yes	Yes	No	Yes	Yes	Yes	Yes	No	Yes	Critically low
Guo 2022	Yes	PY	Yes	PY	Yes	Yes	No	Yes	Yes	No	Yes	Yes	Yes	Yes	Yes	Yes	Low
Hall 2017	Yes	PY	Yes	PY	No	Yes	No	Yes	Yes	No	Yes	Yes	Yes	Yes	No	Yes	Critically low
Hall 2008	Yes	No	Yes	PY	No	No	No	Yes	Yes	No	Yes	Yes	Yes	Yes		Yes	Critically low
Hall 2009	Yes	No	Yes	Yes	No	Yes	No	Yes	Yes	No	Yes	Yes	Yes	Yes	NA	Yes	Critically low
Han 2024	Yes	PY	Yes	PY	Yes	Yes	Yes	Yes	Yes	No	Yes	Yes	Yes	Yes	Yes	Yes	High
Hansen 2020	Yes	PY	Yes	PY	Yes	Yes	No	Yes	Yes	No	Yes	Yes	Yes	Yes	NA	Yes	Low
Hart 2019	Yes	PY	Yes	PY	Yes	Yes	No	Yes	Yes	No	Yes	Yes	Yes	Yes	Yes	Yes	Low
Häuser 2010	Yes	No	Yes	PY	Yes	Yes	No	Yes	Yes	No	Yes	Yes	Yes	Yes	Yes	Yes	Critically low
Hayden 2020	Yes	PY	Yes	PY	No	No	No	Yes	Yes	No	Yes	Yes	Yes	Yes	No	Yes	Critically low
Heidari 2023	Yes	PY	Yes	PY	Yes	Yes	No	Yes	Yes	No	Yes	Yes	Yes	Yes	Yes	Yes	Low

Hernandez-Lucas 2022	Yes	PY	Yes	Yes	Yes	Yes	No	Yes	Yes	No	Yes	Yes	Yes	Yes	NA	Yes	Low
Hernández-Molina 2008	Yes	No	Yes	PY	Yes	Yes	No	Yes	Yes	No	Yes	Yes	Yes	Yes	NA	Yes	Critically low
Holden 2023	Yes	PY	Yes	PY	Yes	No	No	Yes	Yes	No	Yes	Yes	Yes	Yes	Yes	Yes	Low
Holtzman 2013	Yes	No	Yes	PY	No	No	No	Yes	Yes	No	Yes	Yes	Yes	Yes	NA	Yes	Critically low
Hsueh 2021	Yes	PY	Yes	PY	No	Yes	No	Yes	Yes	No	Yes	Yes	Yes	Yes	No	Yes	Critically low
Huang 2023	Yes	PY	Yes	Yes	Yes	Yes	No	Yes	Yes	No	Yes	Yes	Yes	Yes	Yes	Yes	Low
Hu 2021	Yes	PY	Yes	PY	Yes	Yes	No	Yes	Yes	No	Yes	Yes	Yes	Yes	Yes	Yes	Low
Hu 2020	Yes	PY	Yes	PY	Yes	Yes	No	Yes	Yes	No	Yes	Yes	Yes	Yes	NA	Yes	Low
Ji-Ah 2022	Yes	PY	Yes	PY	Yes	No	No	Yes	Yes	No	Yes	Yes	Yes	Yes	No	Yes	Critically low
Jones 2022	Yes	PY	Yes	PY	Yes	Yes	No	Yes	Yes	No	Yes	Yes	Yes	Yes	Yes	Yes	Low
Juhl 2014	Yes	No	Yes	PY	Yes	No	No	Yes	Yes	No	Yes	Yes	Yes	Yes	Yes	Yes	Critically low
Jurado-Castro 2022	Yes	PY	Yes	PY	Yes	Yes	No	Yes	Yes	No	Yes	Yes	Yes	Yes	NA	Yes	Low
Kang 2011	Yes	No	Yes	PY	No	Yes	No	Yes	Yes	No	Yes	Yes	Yes	Yes	NA	Yes	Critically low
Kelley 2011	Yes	No	Yes	PY	Yes	Yes	No	Yes	Yes	No	Yes	Yes	Yes	Yes	Yes	Yes	Critically low
Kim 2019	Yes	PY	Yes	PY	No	No	No	Yes	Yes	No	Yes	Yes	Yes	Yes	NA	Yes	Low
Kim 2020	Yes	PY	Yes	PY	No	No	No	Yes	Yes	No	Yes	Yes	Yes	Yes	NA	Yes	Low
Kong 2016	Yes	PY	Yes	PY	Yes	Yes	No	Yes	Yes	No	Yes	Yes	Yes	Yes	Yes	Yes	Low
Kong 2022	Yes	PY	Yes	PY	No	Yes	No	Yes	Yes	No	Yes	Yes	Yes	Yes	Yes	Yes	Low
Langhorst 2013	Yes	No	Yes	PY	Yes	Yes	No	Yes	Yes	No	Yes	Yes	Yes	Yes	NA	Yes	Critically low
LaTouche 2020	Yes	PY	Yes	PY	Yes	Yes	No	Yes	Yes	No	Yes	Yes	Yes	Yes	Yes	Yes	Low
Lauche 2019	Yes	PY	Yes	PY	Yes	Yes	No	Yes	Yes	No	Yes	Yes	Yes	Yes	No	Yes	Critically low
Lauche 2013	Yes	No	Yes	PY	Yes	Yes	No	Yes	Yes	No	Yes	Yes	Yes	Yes	NA	Yes	Critically low
Lawrence 2017	Yes	Yes	Yes	Yes	Yes	Yes	Yes	Yes	Yes	Yes	Yes	Yes	Yes	Yes	NA	Yes	High
Lezheng 2023	Yes	Yes	Yes	Yes	Yes	Yes	No	Yes	Yes	No	Yes	Yes	Yes	Yes	NA	Yes	Low
Liang 2019	Yes	PY	Yes	PY	Yes	Yes	No	Yes	Yes	No	Yes	Yes	Yes	Yes	NA	Yes	Low

Li 2023	Yes	Yes	Yes	PY	No	No	No	Yes	Yes	No	Yes	Yes	Yes	Yes	No	Yes	Critically low
Lim 2011	Yes	No	Yes	PY	Yes	Yes	No	Yes	Yes	No	Yes	Yes	Yes	Yes	NA	Yes	Critically low
Li 2020	Yes	PY	Yes	PY	Yes	Yes	No	Yes	Yes	No	Yes	Yes	Yes	Yes	Yes	Yes	Low
Liu 2009	Yes	Yes	Yes	Yes	Yes	Yes	Yes	Yes	Yes	Yes	Yes	Yes	Yes	Yes	No	Yes	Low
Li 2009	Yes	No	Yes	PY	Yes	No	No	Yes	Yes	No	Yes	Yes	Yes	Yes	NA	Yes	Critically low
Li 2023	Yes	PY	Yes	PY	No	Yes	No	Yes	Yes	No	Yes	Yes	Yes	Yes	No	Yes	Critically low
Li 2016	Yes	PY	Yes	PY	Yes	Yes	No	Yes	Yes	No	Yes	Yes	Yes	Yes	No	Yes	Critically low
Long 2022	Yes	PY	Yes	PY	No	Yes	No	Yes	Yes	No	Yes	Yes	Yes	Yes	NA	Yes	Low
Luan 2021	Yes	PY	Yes	PY	Yes	Yes	No	Yes	Yes	No	Yes	Yes	Yes	Yes	No	Yes	Critically low
Lu 2024	Yes	PY	Yes	PY	Yes	Yes	No	Yes	Yes	No	Yes	Yes	Yes	Yes	NA	Yes	Low
Luo 2020	Yes	PY	Yes	PY	No	Yes	No	Yes	Yes	No	Yes	Yes	Yes	Yes	No	Yes	Critically low
Mahmoudi 2022	Yes	PY	Yes	Yes	Yes	Yes	No	Yes	Yes	No	Yes	Yes	Yes	Yes	No	Yes	Critically low
Ma 2018	Yes	PY	Yes	PY	Yes	Yes	No	Yes	Yes	No	Yes	Yes	Yes	Yes	NA	Yes	Low
Mao 2024	Yes	PY	Yes	Yes	Yes	Yes	No	Yes	Yes	No	Yes	Yes	Yes	Yes	Yes	Yes	Low
Meender Schäfer 2018	Yes	PY	Yes	PY	Yes	No	No	Yes	Yes	No	Yes	Yes	Yes	Yes	NA	Yes	Low
Miller 2022	Yes	PY	Yes	PY	Yes	Yes	No	Yes	Yes	No	Yes	Yes	Yes	Yes	Yes	Yes	Low
Miyamoto 2013	Yes	No	Yes	PY	Yes	No	No	Yes	Yes	No	Yes	Yes	Yes	Yes	NA	Yes	Critically low
Moseng 2017	Yes	PY	Yes	PY	Yes	Yes	No	Yes	Yes	No	Yes	Yes	Yes	Yes	No	Yes	Critically low
Murillo-García 2018	Yes	PY	Yes	PY	Yes	Yes	No	Yes	Yes	No	Yes	Yes	Yes	Yes	NA	Yes	Low
Nakano 2018	Yes	PY	Yes	PY	Yes	Yes	No	Yes	Yes	No	Yes	Yes	Yes	Yes	NA	Yes	Low
Nunan 2022	Yes	Yes	Yes	Yes	Yes	Yes	Yes	Yes	Yes	Yes	Yes	Yes	Yes	Yes	Yes	Yes	High
Oliveira 2018	Yes	PY	Yes	PY	Yes	Yes	No	Yes	Yes	No	Yes	Yes	Yes	Yes	NA	Yes	Low
Pedersen 2023	Yes	PY	Yes	PY	Yes	Yes	No	Yes	Yes	No	Yes	Yes	Yes	Yes	Yes	Yes	Low
Pereira 2012	Yes	No	Yes	PY	No	No	No	Yes	Yes	No	Yes	Yes	Yes	Yes	NA	Yes	Critically low
Plinsinga 2023	Yes	Yes	Yes	Yes	Yes	Yes	No	Yes	Yes	No	Yes	Yes	Yes	Yes	Yes	Yes	Low

Polaski 2019	Yes	PY	Yes	PY	No	No	No	Yes	Yes	No	Yes	Yes	Yes	Yes	No	Yes	Critically low
Ponzano 2021	Yes	PY	Yes	PY	Yes	Yes	No	Yes	Yes	No	Yes	Yes	Yes	Yes	Yes	Yes	Low
Qian 2020	Yes	PY	Yes	PY	Yes	No	No	Yes	Yes	No	Yes	Yes	Yes	Yes	Yes	Yes	Low
Qiao 2024	Yes	PY	Yes	PY	No	Yes	No	Yes	Yes	No	Yes	Yes	Yes	Yes	Yes	Yes	Low
Qin 2021	Yes	PY	Yes	PY	Yes	Yes	No	Yes	Yes	No	Yes	Yes	Yes	Yes	Yes	Yes	Low
Qin 2019	Yes	PY	Yes	PY	Yes	Yes	No	Yes	Yes	No	Yes	Yes	Yes	Yes	NA	Yes	Low
Quentin 2021	Yes	PY	Yes	PY	Yes	Yes	No	Yes	Yes	No	Yes	Yes	Yes	Yes	Yes	Yes	Low
Ramel 2009	Yes	No	Yes	PY	No	Yes	No	Yes	Yes	No	Yes	Yes	Yes	Yes	No	Yes	Critically low
Roberts 2020	Yes	Yes	Yes	Yes	Yes	Yes	Yes	Yes	Yes	Yes	Yes	Yes	Yes	Yes	NA	Yes	High
Roddy 2005	Yes	No	Yes	PY	No	Yes	No	Yes	Yes	No	Yes	Yes	Yes	Yes	No	Yes	Critically low
Rodríguez-Almagro 2023	Yes	PY	Yes	PY	Yes	Yes	No	Yes	Yes	No	Yes	Yes	Yes	Yes	Yes	Yes	Low
Sánchez-Polán 2023	Yes	PY	Yes	PY	Yes	Yes	No	Yes	Yes	No	Yes	Yes	Yes	Yes	Yes	Yes	Low
Saragiotto 2020	Yes	PY	Yes	PY	Yes	Yes	No	Yes	Yes	No	Yes	Yes	Yes	Yes	No	Yes	Critically low
Sasaki 2022	Yes	PY	Yes	PY	Yes	Yes	No	Yes	Yes	No	Yes	Yes	Yes	Yes	Yes	Yes	Low
Shen 2020	Yes	PY	Yes	PY	Yes	No	No	Yes	Yes	No	Yes	Yes	Yes	Yes	Yes	Yes	Low
Shiri 2018	Yes	PY	Yes	PY	No	No	No	Yes	Yes	No	Yes	Yes	Yes	Yes	Yes	Yes	Low
Sobue 2022	Yes	Yes	Yes	PY	Yes	No	No	Yes	Yes	No	Yes	Yes	Yes	Yes	NA	Yes	Low
Tanaka 2014	Yes	No	Yes	PY	Yes	No	No	Yes	Yes	No	Yes	Yes	Yes	Yes	NA	Yes	Critically low
Tanaka 2013	Yes	No	Yes	PY	Yes	Yes	No	Yes	Yes	No	Yes	Yes	Yes	Yes	No	Yes	Critically low
Tan 2024	Yes	PY	Yes	Yes	Yes	Yes	No	Yes	Yes	No	Yes	Yes	Yes	Yes	Yes	Yes	Low
Tanriverdi 2023	Yes	PY	Yes	PY	Yes	No	No	Yes	Yes	No	Yes	Yes	Yes	Yes	No	Yes	Critically low
Teirlinck 2023	Yes	PY	Yes	PY	Yes	Yes	No	Yes	Yes	No	Yes	Yes	Yes	Yes	Yes	Yes	Low
Umehara 2018	Yes	PY	Yes	PY	Yes	No	No	Yes	Yes	No	Yes	Yes	Yes	Yes	No	Yes	Critically low
Varangot-Reille 2022	Yes	PY	Yes	PY	Yes	Yes	No	Yes	Yes	No	Yes	Yes	Yes	Yes	Yes	Yes	Low
Verville 2023	Yes	PY	Yes	PY	Yes	Yes	No	Yes	Yes	No	Yes	Yes	Yes	Yes	No	Yes	Critically low

Vilarino 2023	Yes	PY	Yes	PY	Yes	Yes	No	Yes	Yes	No	Yes	Yes	Yes	Yes	No	Yes	Critically low
Waller 2014	Yes	No	Yes	PY	Yes	Yes	No	Yes	Yes	No	Yes	Yes	Yes	Yes	No	Yes	Low
Wang 2024	Yes	PY	Yes	PY	No	Yes	No	Yes	Yes	No	Yes	Yes	Yes	Yes	No	Yes	Critically low
Wang Susu 2021	Yes	PY	Yes	PY	Yes	Yes	No	Yes	Yes	No	Yes	Yes	Yes	Yes	No	Yes	Critically low
Wang 2023	Yes	Yes	Yes	PY	No	Yes	No	Yes	Yes	No	Yes	Yes	Yes	Yes	Yes	Yes	Low
Wang 2018	Yes	PY	Yes	PY	Yes	Yes	No	Yes	Yes	No	Yes	Yes	Yes	Yes	Yes	Yes	Low
Wang 2017	Yes	PY	Yes	PY	No	Yes	No	Yes	Yes	No	Yes	Yes	Yes	Yes	NA	Yes	Low
Wei 2024	Yes	Yes	Yes	Yes	Yes	Yes	No	Yes	Yes	No	Yes	Yes	Yes	Yes	NA	Yes	Low
Wei 2015	Yes	PY	Yes	PY	Yes	Yes	No	Yes	Yes	No	Yes	Yes	Yes	Yes	Yes	Yes	Low
Wen 2022	Yes	PY	Yes	PY	Yes	Yes	No	Yes	Yes	No	Yes	Yes	Yes	Yes	Yes	Yes	Low
Wewege 2018	Yes	PY	Yes	PY	Yes	Yes	No	Yes	Yes	No	Yes	Yes	Yes	Yes	NA	Yes	Low
Wu 2022,	Yes	PY	Yes	PY	Yes	Yes	No	Yes	Yes	No	Yes	Yes	Yes	Yes	NA	Yes	Low
Wu 2022	Yes	PY	Yes	PY	No	Yes	No	Yes	Yes	No	Yes	Yes	Yes	Yes	NA	Yes	Low
Yamato 2016	Yes	Yes	Yes	Yes	Yes	Yes	Yes	Yes	Yes	Yes	Yes	Yes	Yes	Yes	NA	Yes	High
Yang 2022	Yes	Yes	Yes	PY	Yes	Yes	No	Yes	Yes	No	Yes	Yes	Yes	Yes	Yes	Yes	Low
Yang 2023	Yes	Yes	Yes	PY	Yes	Yes	No	Yes	Yes	No	Yes	Yes	Yes	Yes	NA	Yes	Low
Yang 2022	Yes	Yes	Yes	PY	Yes	Yes	No	Yes	Yes	No	Yes	Yes	Yes	Yes	NA	Yes	Low
Yan 2013	Yes	No	Yes	PY	Yes	Yes	No	Yes	Yes	No	Yes	Yes	Yes	Yes	NA	Yes	Critically low
Yan 2021	Yes	PY	Yes	PY	Yes	Yes	No	Yes	Yes	No	Yes	Yes	Yes	Yes	Yes	Yes	Low
Ye 2022	Yes	Yes	Yes	PY	Yes	Yes	No	Yes	Yes	No	Yes	Yes	Yes	Yes	No	Yes	Critically low
Ye 2020	Yes	PY	Yes	PY	Yes	Yes	No	Yes	Yes	No	Yes	Yes	Yes	Yes	NA	Yes	Low
Yilmaz 2023	Yes	PY	Yes	PY	Yes	No	No	Yes	Yes	No	Yes	Yes	Yes	Yes	NA	Yes	Low
Zeng 2020	Yes	PY	Yes	PY	Yes	Yes	No	Yes	Yes	No	Yes	Yes	Yes	Yes	NA	Yes	Low
Zhang 2022	Yes	PY	Yes	PY	No	No	No	Yes	Yes	No	Yes	Yes	Yes	Yes	NA	Yes	Low
Zhang 2023	Yes	PY	Yes	PY	No	No	No	Yes	Yes	No	Yes	Yes	Yes	Yes	NA	Yes	Low
Zhang 2024	Yes	Yes	Yes	PY	Yes	Yes	No	Yes	Yes	No	Yes	Yes	Yes	Yes	Yes	Yes	Low
Zhang 2023	Yes	Yes	Yes	PY	Yes	Yes	No	Yes	Yes	No	Yes	Yes	Yes	Yes	Yes	Yes	Low
Zhang 2023	Yes	Yes	Yes	PY	Yes	Yes	No	Yes	Yes	No	Yes	Yes	Yes	Yes	Yes	Yes	Low
Zhang 2023	Yes	Yes	Yes	PY	No	Yes	No	Yes	Yes	No	Yes	Yes	Yes	Yes	Yes	Yes	Low
Zhang 2017	Yes	PY	Yes	PY	Yes	Yes	No	Yes	Yes	No	Yes	Yes	Yes	Yes	NA	Yes	Low

Zhang 2019	Yes	Yes	Yes	PY	Yes	Yes	No	Yes	Yes	No	Yes	Yes	Yes	Yes	Yes	Yes	Low
Zhang 2019	Yes	Yes	Yes	PY	Yes	Yes	No	Yes	Yes	No	Yes	Yes	Yes	Yes	Yes	Yes	Low
Zhou 2019	Yes	PY	Yes	PY	Yes	Yes	No	Yes	Yes	No	Yes	Yes	Yes	Yes	Yes	Yes	Low
Zhu 2020	Yes	PY	Yes	PY	Yes	No	No	Yes	Yes	No	Yes	Yes	Yes	Yes	Yes	Yes	Low
Zou 2019	Yes	No	Yes	PY	Yes	Yes	No	Yes	Yes	No	Yes	Yes	Yes	Yes	Yes	Yes	Critically low

AMSTAR-2 Items
1. Did the research questions and inclusion criteria for the review include the components of PICO?
2. Did the report of the review contain an explicit statement that the review methods were established prior to the conduct of the review and did the report justify any significant deviations from the protocol?
3. Did the review authors explain their selection of the study designs for inclusion in the review?
4. Did the review authors use a comprehensive literature search strategy?
5. Did the review authors perform study selection in duplicate?
6. Did the review authors perform data extraction in duplicate?
7. Did the review authors provide a list of excluded studies and justify the exclusions?
8. Did the review authors describe the included studies in adequate detail?
9. Did the review authors use a satisfactory technique for assessing the risk of bias (RoB) in individual studies that were included in the review?
10. Did the review authors report on the sources of funding for the studies included in the review?
11. If meta-analysis was performed did the review authors use appropriate methods for statistical combination of results?
12. If meta-analysis was performed, did the review authors assess the potential impact of RoB in individual studies on the results of the meta-analysis or other evidence synthesis?
13. Did the review authors account for RoB in individual studies when interpreting/ discussing the results of the review?
14. Did the review authors provide a satisfactory explanation for, and discussion of, any heterogeneity observed in the results of the review?
15. If they performed quantitative synthesis did the review authors carry out an adequate investigation of publication bias (small study bias) and discuss its likely impact on the results of the review?
16. Did the review authors report any potential sources of conflict of interest, including any funding they received for conducting the review?

Supplementary content 5. Overall meta-analysis results for pain.

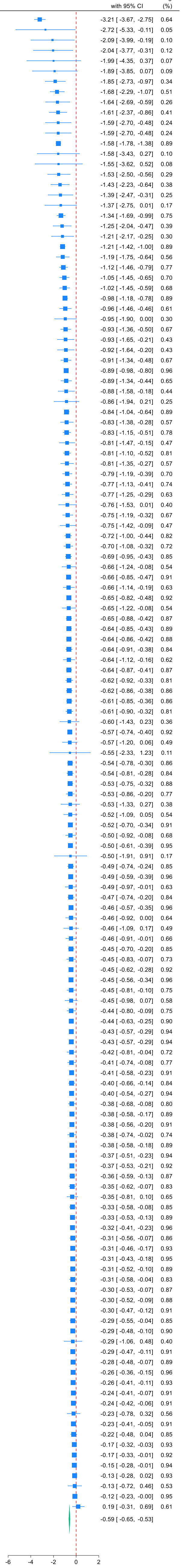

Study	Effect size with 95% CI	Weight (%)
Hall 2017 (Intermediate term)	-3.21 [-3.67, -2.75]	0.64
Lim 2011	-2.72 [-5.33, -0.11]	0.05
Kim 2019	-2.09 [-3.99, -0.19]	0.10
Sobue 2022	-2.04 [-3.77, -0.31]	0.12
Pereira 2012	-1.99 [-4.35, 0.37]	0.07
Busch 2013	-1.89 [-3.85, 0.07]	0.09
Hall 2017 (Short term)	-1.85 [-2.73, -0.97]	0.34
Wang 2023 (Low back pain)	-1.68 [-2.29, -1.07]	0.51
Murillo-García 2018	-1.64 [-2.69, -0.59]	0.26
Barros Dos Santos 2021	-1.61 [-2.37, -0.86]	0.41
Shen 2020	-1.59 [-2.70, -0.48]	0.24
Qian 2020	-1.59 [-2.70, -0.48]	0.24
Zhang 2022	-1.58 [-1.78, -1.38]	0.89
Hall 2017 (Short term)	-1.58 [-3.43, 0.27]	0.10
Fernandopulle 2017 (Long term)	-1.55 [-3.62, 0.52]	0.08
Wei 2024	-1.53 [-2.50, -0.56]	0.29
Wu 2022B (Headache frequency)	-1.43 [-2.23, -0.64]	0.38
Yan 2021	-1.39 [-2.47, -0.31]	0.25
Wu 2022B (Pain intensity)	-1.37 [-2.75, 0.01]	0.17
Couto 2022	-1.34 [-1.69, -0.99]	0.75
LaTouche 2020	-1.25 [-2.04, -0.47]	0.39
Long 2022	-1.21 [-2.17, -0.25]	0.30
Yilmaz 2023 (Labour)	-1.21 [-1.42, -1.00]	0.89
Busch 2002 (Tender points)	-1.19 [-1.75, -0.64]	0.56
Kong 2022	-1.12 [-1.46, -0.79]	0.77
Zhang 2023A	-1.05 [-1.45, -0.65]	0.70
Varangot-Reille 2022 (Headache)	-1.02 [-1.45, -0.59]	0.68
Wang 2018	-0.98 [-1.18, -0.78]	0.89
De Almeida 2024	-0.96 [-1.46, -0.46]	0.61
Al-Mhanna 2022	-0.95 [-1.90, 0.00]	0.30
Pedersen 2023	-0.93 [-1.36, -0.50]	0.67
Gilliam 2023 (Short term)	-0.93 [-1.65, -0.21]	0.43
Lu 2024	-0.92 [-1.64, -0.20]	0.43
Albuquerque 2022	-0.91 [-1.34, -0.48]	0.67
Quentin 2021	-0.89 [-0.98, -0.80]	0.96
Liang 2019	-0.89 [-1.34, -0.44]	0.65
Cheng 2019	-0.88 [-1.58, -0.18]	0.44
Ye 2020	-0.86 [-1.94, 0.21]	0.25
Miller 2022	-0.84 [-1.04, -0.64]	0.89
Kong 2016 (Osteoporosis)	-0.83 [-1.38, -0.28]	0.57
Du 2023	-0.83 [-1.15, -0.51]	0.78
Busch 2007 (Pain)	-0.81 [-1.47, -0.15]	0.47
Kong 2016 (Low back pain)	-0.81 [-1.10, -0.52]	0.81
Hart 2019	-0.81 [-1.35, -0.27]	0.57
Kang 2011	-0.79 [-1.19, -0.39]	0.70
Zhang 2017	-0.77 [-1.13, -0.41]	0.74
Tanaka 2013 (VAS)	-0.77 [-1.25, -0.29]	0.63
Busch 2007 (Tender points)	-0.76 [-1.53, 0.01]	0.40
Lauche 2019	-0.75 [-1.19, -0.32]	0.67
Hernandez-Lucas 2022	-0.75 [-1.42, -0.09]	0.47
Lauche 2013 (Short term)	-0.72 [-1.00, -0.44]	0.82
Biswas 2024	-0.70 [-1.08, -0.32]	0.72
Hu 2021	-0.69 [-0.95, -0.43]	0.85
Fan 2024	-0.66 [-1.24, -0.08]	0.54
Hall 2017 (Short term)	-0.66 [-0.85, -0.47]	0.91
Sánchez-Polán 2023	-0.66 [-1.14, -0.19]	0.63
Kong 2016 (Chronic pain)	-0.65 [-0.82, -0.48]	0.92
Umehara 2018 (Post-operative)	-0.65 [-1.22, -0.08]	0.54
Qiao 2024	-0.65 [-0.88, -0.42]	0.87
Wang 2023 (Fibromyalgia)	-0.64 [-0.85, -0.43]	0.89
Wen 2022	-0.64 [-0.86, -0.42]	0.88
Zhang 2019	-0.64 [-0.91, -0.38]	0.84
Varangot-Reille 2022 (Migraine)	-0.64 [-1.12, -0.16]	0.62
Wang 2023 (Chronic musculoskeletal disorders)	-0.64 [-0.87, -0.41]	0.87
Da Silva 2022	-0.62 [-0.92, -0.33]	0.81
Holtzman 2013	-0.62 [-0.86, -0.38]	0.86
Li 2020	-0.61 [-0.85, -0.36]	0.86
Ji-Ah 2022	-0.61 [-0.90, -0.32]	0.81
Gilliam 2023 (Long term)	-0.60 [-1.43, 0.23]	0.36
Tanaka 2014	-0.57 [-0.74, -0.40]	0.92
Busch 2002 (Pain)	-0.57 [-1.20, 0.06]	0.49
Yilmaz 2023 (Pragnancy)	-0.55 [-2.33, 1.23]	0.11
Kong 2016 (Osteoarthritis)	-0.54 [-0.78, -0.30]	0.86
Duan 2022	-0.54 [-0.81, -0.28]	0.84
Chen 2016	-0.53 [-0.75, -0.32]	0.88
Bertozzi 2013 (Short term)	-0.53 [-0.86, -0.20]	0.77
Oliveira 2018 (Intermediate term)	-0.53 [-1.33, 0.27]	0.38
Kong 2016 (Fibromyalgia)	-0.52 [-1.09, 0.05]	0.54
Roddy 2005 (Aerobic)	-0.52 [-0.70, -0.34]	0.91
Mahmoudi 2022	-0.50 [-0.92, -0.08]	0.68
Juhl 2014	-0.50 [-0.61, -0.39]	0.95
Oliveira 2018 (Short term)	-0.50 [-1.91, 0.91]	0.17
Rodríguez-Almagro 2023	-0.49 [-0.74, -0.24]	0.85
Fransen 2015	-0.49 [-0.59, -0.39]	0.96
Wu 2022A	-0.49 [-0.97, -0.01]	0.63
Zhang 2024	-0.47 [-0.74, -0.20]	0.84
Tan 2024	-0.46 [-0.57, -0.35]	0.96
Demaneuf 2019	-0.46 [-0.92, 0.00]	0.64
Hall 2017 (Long term)	-0.46 [-1.09, 0.17]	0.49
Ye 2022	-0.46 [-0.91, -0.01]	0.66
Yan 2013	-0.45 [-0.70, -0.20]	0.85
Bertozzi 2013 (Intermediate term)	-0.45 [-0.83, -0.07]	0.73
Plinsinga 2023	-0.45 [-0.62, -0.28]	0.92
Guo 2022	-0.45 [-0.56, -0.34]	0.96
Ramel 2009	-0.45 [-0.81, -0.10]	0.75
Hall 2017 (Intermediate term)	-0.45 [-0.98, 0.07]	0.58
Aladro-Gonzalvo 2013	-0.44 [-0.80, -0.09]	0.75
Lezheng 2023	-0.44 [-0.63, -0.25]	0.90
Tanaka 2013 (WOMAC)	-0.43 [-0.57, -0.29]	0.94
Li 2016	-0.43 [-0.57, -0.29]	0.94
Wewege 2018	-0.42 [-0.81, -0.04]	0.72
Chen 2021 (Hip pain)	-0.41 [-0.74, -0.08]	0.77
Kim 2020	-0.41 [-0.58, -0.23]	0.91
Huang 2023	-0.40 [-0.66, -0.14]	0.84
Gámez-Iruela 2024	-0.40 [-0.54, -0.27]	0.94
Hernández-Molina 2008	-0.38 [-0.68, -0.08]	0.80
Austin 2024	-0.38 [-0.58, -0.17]	0.89
Fransen 2014	-0.38 [-0.56, -0.20]	0.91
Hsueh 2021	-0.38 [-0.74, -0.02]	0.74
Mao 2024	-0.38 [-0.58, -0.18]	0.89
Zou 2019	-0.37 [-0.51, -0.23]	0.94
Kelley 2011	-0.37 [-0.53, -0.21]	0.92
Wang 2023 (Osteoarthritis)	-0.36 [-0.59, -0.13]	0.87
Han 2024	-0.35 [-0.62, -0.07]	0.83
Langhorst 2013	-0.35 [-0.81, 0.10]	0.65
Verville 2023	-0.33 [-0.58, -0.08]	0.85
Ariie 2023	-0.33 [-0.53, -0.13]	0.89
Roddy 2005 (Resistance)	-0.32 [-0.41, -0.23]	0.96
Baillet 2010	-0.31 [-0.56, -0.07]	0.86
Häuser 2010	-0.31 [-0.46, -0.17]	0.93
Barnet-Hepples 2024	-0.31 [-0.43, -0.18]	0.95
Zhang 2023B	-0.31 [-0.52, -0.10]	0.89
Meender Schäfer 2018 (Short term)	-0.31 [-0.58, -0.04]	0.83
Meender Schäfer 2018 (Long term)	-0.30 [-0.53, -0.07]	0.87
Luo 2020	-0.30 [-0.52, -0.09]	0.88
Liu 2009	-0.30 [-0.47, -0.12]	0.91
Tanriverdi 2023	-0.29 [-0.55, -0.04]	0.85
Chen 2021 (Knee pain)	-0.29 [-0.48, -0.10]	0.90
Lauche 2013 (Long term)	-0.29 [-1.06, 0.48]	0.40
Diez-Buil 2024	-0.29 [-0.47, -0.11]	0.91
Yang 2023	-0.28 [-0.48, -0.07]	0.89
Ponzano 2021	-0.26 [-0.36, -0.15]	0.96
Waller 2014	-0.26 [-0.41, -0.11]	0.93
Björk 2022	-0.24 [-0.41, -0.07]	0.91
Moseng 2017	-0.24 [-0.42, -0.06]	0.91
Roberts 2020	-0.23 [-0.78, 0.32]	0.56
Teirlinck 2023	-0.23 [-0.41, -0.05]	0.91
Umehara 2018 (Pre-operative)	-0.22 [-0.48, 0.04]	0.85
Nakano 2018	-0.17 [-0.32, -0.03]	0.93
Hall 2008	-0.17 [-0.33, -0.01]	0.92
Sasaki 2022	-0.15 [-0.28, -0.01]	0.94
Wang Susu 2021	-0.13 [-0.28, 0.02]	0.93
Nunan 2022	-0.13 [-0.72, 0.46]	0.53
Fisseha 2016	-0.12 [-0.23, -0.00]	0.95
Fernandopulle 2017 (Intermediate term)	0.19 [-0.31, 0.69]	0.61
**Overall**	-0.59 [-0.65, -0.53]	

Heterogeneity: τ^2 = 0.10, I^2 = 88.18%, H^2 = 8.46

Test of $\theta_i = \theta_j$: Q(144) = 852.17, p = 0.00

Test of θ = 0: z = -18.42, p = 0.00

Random-effects REML model

Supplementary content 6. Funnel plots for pain.

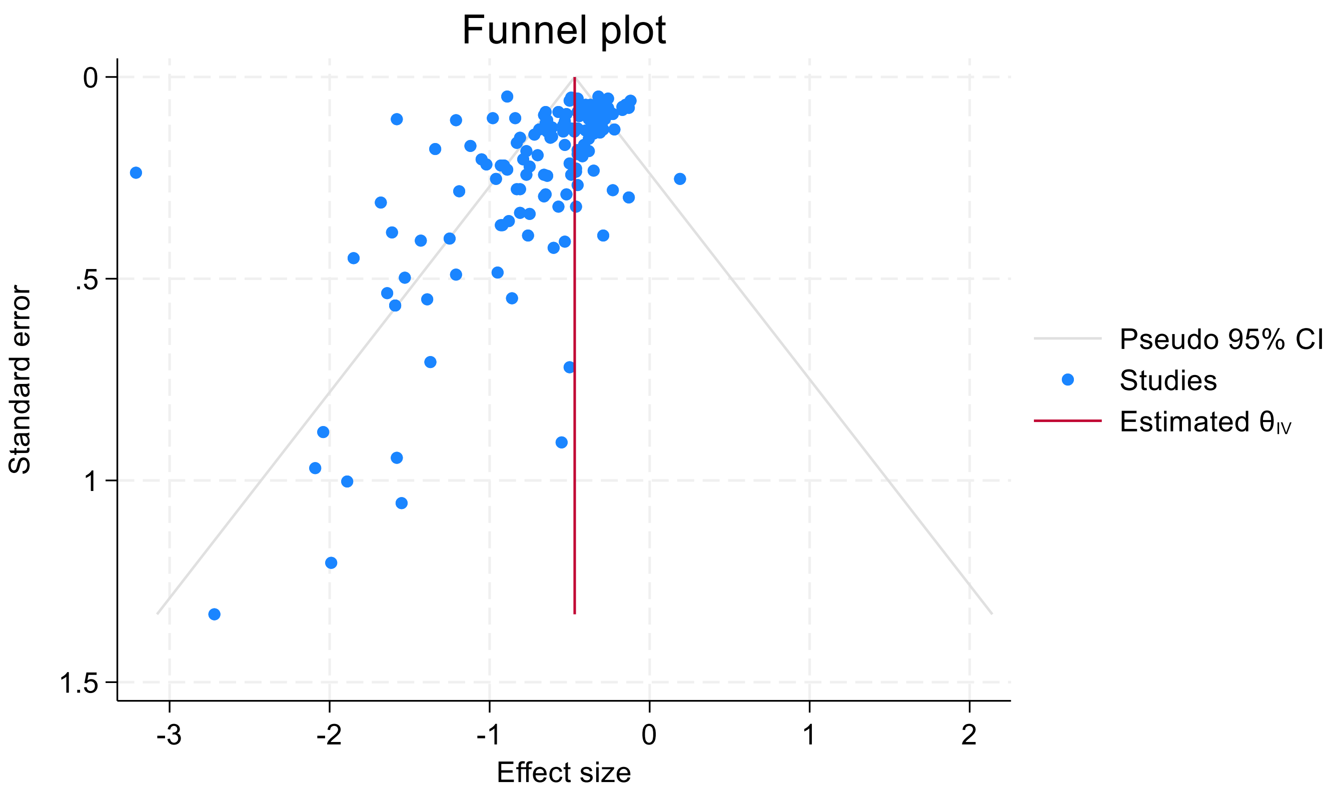

Supplementary content 7. Subgroup effects for physical activity parameters for pain

	Studies	SMD	95% CI	I^2 (%)	Test of subgroup differences
**Pain type**					Q_b (1) = 2.76, p = 0.10
Acute	21	-0.87	-1.21, -0.53	95.06	
Chronic	90	-0.57	-0.64, -0.51	98.69	
**Intervention duration**					Q_b (1) = 4.61, p = 0.03
Less than 12 weeks	10	-0.59	-0.78, -0.41	35.31	
12 weeks or more	24	-0.37	-0.45, -0.29	32.78	
**Exercise intensity**					Q_b (1) = 17.80, p < 0.01
Low	55	-0.77	-0.90, -0.64	89.54	
Moderate-to-vigorous	90	-0.47	-0.52, -0.41	77.14	
**Sessions per week**					Q_b (1) = 0.13, p = 0.72
1 to 2	5	-0.53	-0.75, -0.32	35.55	
3 or more	4	-0.47	-0.75, -0.18	62.33	
**Session duration**					Q_b (1) = 1.25, p = 0.26
60 minutes or less	3	-0.68	-1.14, -0.23	48.65	
More than 60 minutes	2	-0.41	-0.58, -0.23	0.00	
**Weekly duration**					Q_b (1) = 5.48, p = 0.02
Less than 120 minutes	1	-0.90	-1.8, 0.00	-	
120 minutes or more	2	0.58	-0.27, 1.42	0.00	
**AMSTAR-2 score**					Q_b (2) = 18.73, p<0.01
Critically low	51	-0.57	-0.70, -0.43	91.7	
Low	81	-0.64	-0.71, -0.56	86.3	
High	12	-0.40	-0.48, -0.33	17.9	

Supplementary content 8. Overview of GRADE ratings for pain.

Outcome	Effect Size (SMD)	Risk of Bias	Inconsistency	Indirectness	Imprecision	Publication Bias	GRADE Certainty Rating
Pain	-0.59 (95% CI, -0.65 to -0.53)	Serious (many included reviews had low or critically low AMSTAR-2 scores)	Moderate (I^2 = 88.2%)	Low (evidence directly applies to target populations with pain)	Low (large number of RCTs and narrow confidence intervals)	Likely (funnel plot asymmetry suggests small-study effects, but adjusted effect size remained robust)	**Moderate**
